# Structural Effect of Rhenium‐ and Iridium‐Complex Liposome Composition on Their Selectivity for Antimicrobial Photodynamic Therapy

**DOI:** 10.1002/smsc.202300131

**Published:** 2023-12-14

**Authors:** Giulia Kassab, Neha Manav, Layla Pires, Miffy H. Y. Cheng, Yulin Mo, Hilde H. Buzzá, Iti Gupta, Juan Chen, Gang Zheng

**Affiliations:** ^1^ Princess Margaret Cancer Centre University Health Network Toronto M5G 1L7 Canada; ^2^ Department of Medical Biophysics University of Toronto Toronto M5G 1L7 Canada; ^3^ Department of Chemistry Indian Institute of Technology Gandhinagar Gandhinagar 382355 Gujarat India; ^4^ Institute of Medical Science University of Toronto Toronto M5S 1A8 Canada; ^5^ Institute of Physics Pontificia Universidad Católica de Chile 7820436 Santiago Chile

**Keywords:** infections, iridium, liposomes, photodynamic therapy, rhenium

## Abstract

Antimicrobial photodynamic therapy (aPDT) is an alternative to antibiotics that has potential for the treatment of chronic skin wounds, but requires improved, highly selective photosensitizer systems. Rhenium (Re)‐complex‐ and iridium (Ir)‐complex‐based phospholipid conjugates, as PDT‐functional building blocks for liposomes, are presented, and varying structural components and proportion of compounds are explored, including adjusting the cholesterol and polyethylene glycol (PEG)‐lipid contents, incorporating ethylenediaminetetraacetic acid (EDTA)‐lipid, and introducing the cationic lipid 1,2‐dioleoyl‐3‐trimethylammonium propane (DOTAP) to enhance their efficacy and selectivity in aPDT. Ir/Re‐liposomes have nanostructurally enhanced photoactivity compared to monomeric Ir/Re‐lipids. Ir‐liposomes exhibit stronger light absorption and higher emission generation (>threefold) than Re‐liposomes, resulting in superior efficacy against *Staphylococcus aureus* while maintaining better tolerability toward host cells. Formulations with higher cholesterol (40 mol%) and PEG‐lipid (5%) content demonstrate increased potency against *S. aureus*. The incorporation of EDTA‐lipid significantly enhances aPDT efficacy but also increases toxicity toward host cells. Incorporation of DOTAP alters the nanoparticles’ surface charge, potentially improving their interaction with bacterial walls, but negatively impacts their stability, leading to aggregation of the nanoparticles. Ir‐HC demonstrates ideal characteristics (effectiveness, selectivity, and stability) for aPDT under the tested conditions, indicating the importance of the structural design of Re‐ and Ir‐complex liposomes for their selectivity in aPDT.

## Graphical abstract

Innovative rhenium‐complex and iridium‐complex amphiphilic lipids are presented as building blocks for eight different liposome formulations for antimicrobial photodynamic therapy against wound infections. Following a comprehensive characterization of the nanostructures and their optical properties, their effect on bacteria and host cells is tested in vitro and the impact of structural changes in the efficacy and selectivity is discussed.

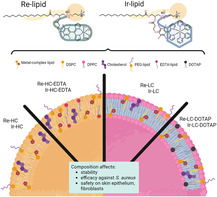

## 1 Introduction

Chronic and acute skin wound infections are a significant cause of morbidity and mortality worldwide. They are mostly caused by bacteria (mainly *Staphylococcus aureus* and *Streptococcus* species) and thus treated with antibiotics, but treatment often fails due to incorrect pathogen identification or antimicrobial resistance.^[^ ^1^, ^2^ ^]^ Additionally, the treatment of chronic skin wounds involves further challenges than the one of acute ones. First, chronic inflammation directly hinders the wound healing process.^[^ ^3^ ^]^ Second, the microbial microenvironment of chronic wounds is more mature, and often includes the extracellular matrix and persister cells from biofilms, making it much harder to treat.^[^ ^4^ ^]^ Finally, due to the presented challenges, chronic wound patients often need recurrent antibiotic treatments, which lead to a selection of resistance, making the antibiotics less and less effective after each treatment.^[^ ^4^ ^]^

Antimicrobial photodynamic therapy (aPDT) is a broad‐spectrum, resistance‐avoidant alternative to antibiotics.^[^ ^5^ ^]^ It is based on the use of photosensitizers, which are molecules capable of generating singlet oxygen and other reactive oxygen species (ROS) after absorbing light at specific wavelengths, leading to cell death. The combined specificity of photosensitizer design and limited light exposure can be used to selectively kill cancer cells (in this case known as just photodynamic therapy or PDT), or pathogens including bacteria, virus, and fungi (aPDT). APDT has been previously proposed for the treatment of skin infections and it has shown significant bacterial killing and wound healing enhancement in preclinical models, but clinical results remain inconsistent.^[^ ^6^, ^7^, ^8^ ^]^

Metal complexes display optoelectronic properties that can be ideal for photodynamic applications, and have gained attention as potential photosensitizers over the last 5 years.^[^ ^9^ ^]^ In particular, rhenium (Re) complexes show lower heavy‐metal toxicity than other metals and have shown interesting preclinical PDT and aPDT results.^[^ ^10^, ^11^, ^12^ ^]^ Iridium (Ir) complexes are known to have fluorescence and phosphorescence that are quenched in the presence of molecular oxygen.^[^ ^13^ ^]^ Thus, some of them have been previously investigated for antitumor and antimicrobial PDT applications.^[^ ^14^, ^15^ ^]^ However, Re and Ir complexes do not show enough solubility in aqueous media to be compatible with biological applications, and require modifications such as the addition of ligands.^[^ ^13^, ^14^, ^16^ ^]^

Liposomal formulations can be designed to overcome solubility issues, as well as increase delivery and selectivity, and have been associated with impactful advances in the field of antimicrobials.^[^ ^17^ ^]^ Recently, an Ir‐complex‐based liposome was shown to have antitumor activity in preclinical models.^[^ ^18^ ^]^ Thus, we have designed two amphiphilic Re‐ and Ir‐complex lipids with photosensitizer properties and integrated them into liposomes for aPDT of chronic wound infections. Additionally, as selectivity is so essential for the success of chronic wound treatments, we have prepared multiple liposomal formulations with varying membrane components and proportions of active compounds and investigated their cytotoxicity against two model pathogens (*S. aureus* and *Escherichia coli*) and two healthy human cell lines (HDFn fibroblasts and HaCat keratinocytes). We found that altering the composition of the liposomes significantly changes their efficacy and toxicity.

## 2 Results

### 2.1 Re‐ and Ir‐Lipid Synthesis

**Figure 1:**
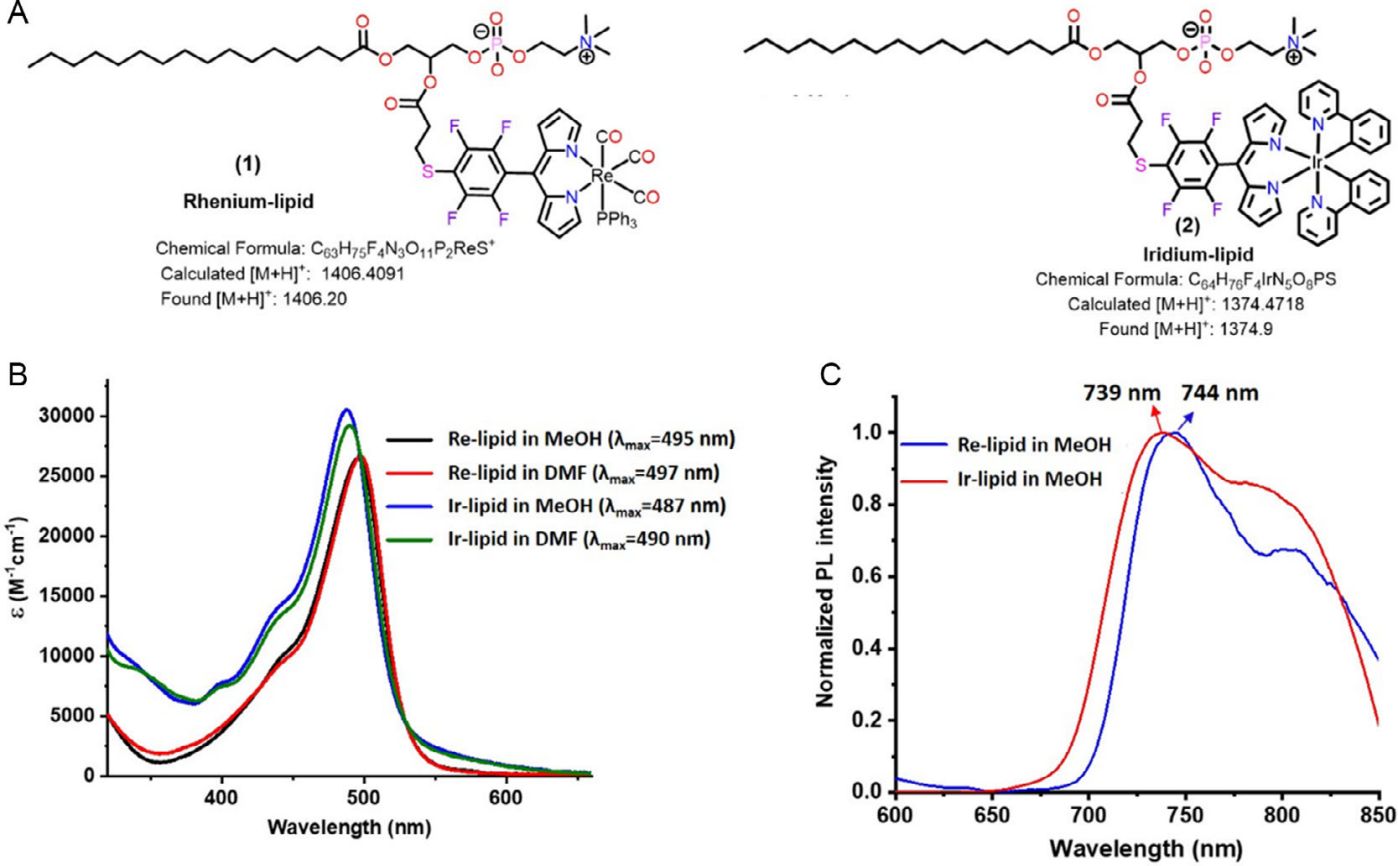
A) Chemical structures of Re‐lipid conjugate (compound **1**) and Ir‐lipid conjugate (compound **2**); B) absorbance spectra of Re‐ and Ir‐lipid in MeOH and DMF; and C) normalized emission of Re‐ and Ir‐lipid in MeOH.

The desired metal–lipid conjugates, Re complex lipid (Re‐lipid, compound **1**) and Ir complex lipid (Ir‐lipid, compound **2**) (see their molecular structures in **Figure** **1**A), were prepared following the synthesis process illustrated in Scheme S1, Supporting Information. Briefly, Re dipyrrinate (compound **3**) and Ir dipyrrinate (compound **5**) were first synthesized following a previously published method.^[^ ^19^, ^20^ ^]^ They were then reacted with mercaptopropionic acid in the presence of a base through a nucleophilic substitution reaction on the *para*‐position of pentafluorophenyl. This reaction yielded Re dipyrrinate acid (compound **4**) and Ir dipyrrinate acid (compound **6**) with a yield of ≈90%. Subsequently, these metal dipyrrinate acids were conjugated with 1‐palmitoyl‐2‐hydroxy‐*sn*‐glycero‐3‐phosphocholine (16:0 LysoPC) at room temperature in chloroform for 5 and 4 days, respectively. The pure Re‐lipid (compound **1**) and Ir‐lipid (compound **2**) were obtained after silica gel column chromatography, with yields of 18% and 20%, respectively. The lipid conjugates were confirmed by electrospray‐ionization mass spectroscopy (ESI‐MS) and nuclear magnetic resonance (NMR), including ^1^H, ^13^C, ^19^F, and ^31^P (Figure S10–S19, Supporting Information).

### 2.2 Optical Properties and Characterization of Re‐ and Ir‐Lipid

Absorbance measurements of Re‐ and Ir‐lipid were performed in methanol (MeOH) and dimethylformamide (DMF) (Figure 1B). The absorbance peak maxima for Re‐ and Ir‐lipid in MeOH were 495 and 487 nm, with molar extinction coefficients of ≈26 500 and ≈30 500 m ^−1^ cm^−1^, respectively. In DMF, the peak maxima were at 497 and 490 nm, with molar extinction coefficients of 26 700 and 29 200 m ^−1^ cm^−1^, respectively (Figure 1B and S20A,B, Supporting Information). These strong absorbance bands at ≈ 490 nm are predominantly due to the π–π* transition of the dipyrrin ligand. The emission spectra were assessed in MeOH, and the observed emission maxima were at 745 nm for Re‐lipid, and 740 nm for Ir‐lipid (Figure 1C). The observed absorbance and emission spectra of both Re‐ and Ir‐lipid are similar to those of their parent compounds Re dipyrrinate (compound **3**) and Ir dipyrrinate (compound **5**), respectively.^[^ ^19^, ^20^ ^]^

### 2.3 Re‐ and Ir‐Lipid‐Based Liposome Formulation and Characterization

Re‐ and Ir‐lipid were incorporated into liposomes following a previously described method^[^ ^21^ ^]^ to obtain various formulations with varying lipid components (**Table** **1**). In summary, four main formulations of liposome were synthesized for each metal complex. The first formulation design was based on a successful formulation of porphyrin‐based liposome for cancer PDT,^[^ ^22^ ^]^ which consists of 40% cholesterol and 5% 1,2‐distearoyl‐sn‐glycero‐3‐phosphoethanolamine‐*N*‐[methoxy(polyethylene glycol)‐2000] (PEG‐lipid). Building upon this formulation, we designed and synthesized Re‐HC and Ir‐HC liposomes (HC indicating high cholesterol content) containing 40% cholesterol, 5% PEG‐lipid, 25% Re‐lipid or Ir‐lipid, and 30% distearoylphosphatidylcholine (DSPC) as the base lipid. The second formulation, Re‐HC‐ethylenediaminetetraacetic acid (EDTA) and Ir‐HC‐EDTA, incorporated 33% EDTA‐lipid into the Re/Ir‐HC formulation replacing the base lipid DSPC. This design was based on our recent discovery of incorporating EDTA‐lipid into liposomal nanoparticles, enabling the fluidization of the cell membrane in a detergent‐like manner to promote intramembrane uptake of nanoparticles.^[^ ^22^ ^]^ The third formulation had a lower content of cholesterol (17%) and PEG‐lipid (2%), with dipalmitoylphosphatidylcholine (DPPC) as the base lipid instead of DSPC. These formulations were named Re‐LC and Ir‐LC, where LC indicates the low cholesterol content. The last formulation incorporated the cationic 1,2‐dioleoyl‐3‐trimethylammonium propane (DOTAP)‐lipid into the Re‐LC and Ir‐LC formulations to enhance membrane penetration. These formulations were termed Re‐LC‐DOTAP and Ir‐LC‐DOTAP.

**Table 1:** Molar percent composition and characterization of the studied liposomal formulations

Components	Re‐baseda)				Ir‐based			
	Re‐HC	Re‐HC‐EDTA	Re‐LC	Re‐LC‐DOTAP	Ir‐HC	Ir‐HC‐EDTA	Ir‐LC	Ir‐LC‐DOTAP
Re‐lipid (**1**):	25%	20%	21%	20%	–	–	–	–
Ir‐lipid (**2**):	–	–	–	–	25%	20%	21%	20%
Cholesterol:	40%	30%	17%	17%	40%	30%	17%	17%
PEG‐lipid:	5%	5%	2%	2%	5%	5%	2%	2%
EDTA‐lipid:	–	33%	–	–	–	33%	–	–
DOTAP:	–	–	–	20%	–	–	–	20%
DPPC:	–	–	60%	41%	–	–	60%	41%
DSPC:	30%	11%	–	–	30%	11%	–	–
Properties								
Size [nm]	106.4 ± 0.8	84.4 ± 0.3	130.2 ± 0.5	109.8 ± 0.8	122.2 ± 0.6	110.5 ± 1.0	109.7 ± 1.2	157.2 ± 0.9
Polydispersity index	0.107 ± 0.018	0.160 ± 0.016	0.042 ± 0.026	0.129 ± 0.010	0.126 ± 0.007	0.122 ± 0.020	0.102 ± 0.020	0.201 ± 0.013
Zeta potential [mV]	−3.73 ± 0.64	−9.53 ± 1.97	−4.10 ± 0.27	0.990 ± 0.559	−2.73 ± 0.38	−4.87 ± 0.99	−3.17 ± 0.85	1.65 ± 0.21

a) PEG‐lipid, DSPE‐mPEG 2000; EDTA‐lipid, EDTA‐monohexadecylamide lipid;^[^ ^22^ ^]^ DOTAP, 1,2‐dioleoyl‐3‐trimethylammonium‐propane; DPPC, dipalmitoylphosphatidylcholine; DSPC, distearoylphosphatidylcholine.

**Figure 2:**
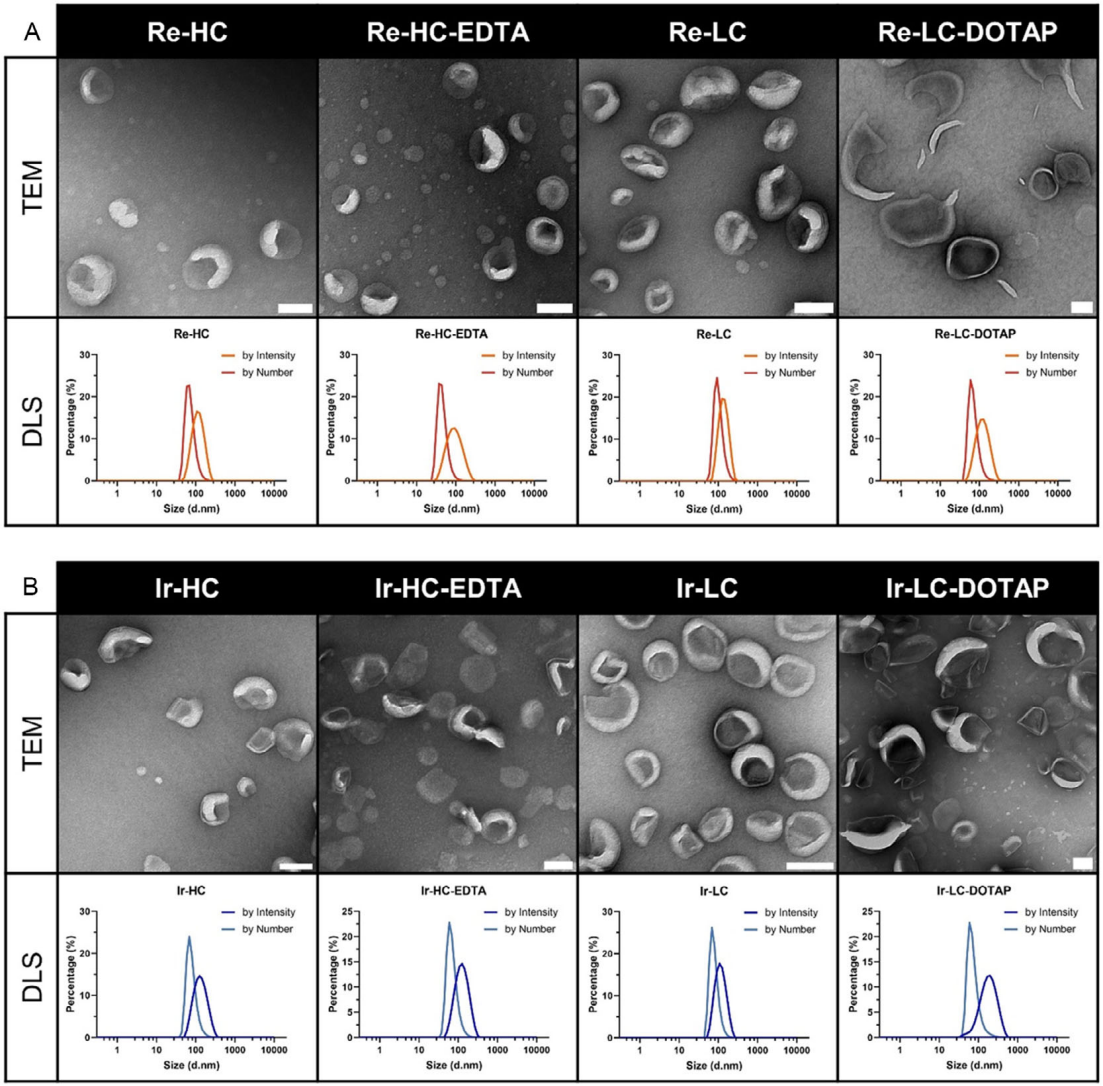
A,B) Characterization of the Re‐complex‐based (A) and Ir‐complex‐based (B) liposomal formulations for TEM imaging, the white bars correspond to 100 nm; for DLS, the size distribution is shown by both intensity and number (representative of three measurements).

The obtained liposomes remained stable under storage conditions (4 °C), maintaining a dynamic light scattering (DLS) size range of ≈80‐150 nm in diameter with monodispersity (polydispersity index [PDI]: 0.04–0.20) (Table 1 and **Figure** **2**). Zeta potential measurements showed that Re‐HC, Ir‐HC, Re‐LC, and Ir‐LC had minimally negative surface charge in phosphate‐buffered saline (PBS) (Table 1). However, an increased negative surface charge was observed when incorporating the negatively charged EDTA‐lipid proportion in Re‐HC‐EDTA and Ir‐HC‐EDTA liposomes. On the other hand, Re‐LC‐DOTAP and Ir‐LC‐DOTAP liposomes exhibited a slightly positive surface charge in PBS. Transmission electron microscopy (TEM) further revealed well‐dispersed spherical vesicles for the six negatively charged formulations: Re‐HC, Ir‐HC, Re‐LC, Ir‐LC, Re‐HC‐EDTA, and Ir‐HC‐EDTA (Figure 2). The positively charged Re‐LC‐DOTAP and Ir‐LC‐DOTAP showed relatively large sizes in DLS size measurement and some elongated vehicle morphologies were observed, likely due to interparticle electronic interactions between the positively charged DOTAP lipid and the negatively charged metal complex‐lipid.

### 2.4 Optical Properties of Re‐ and Ir‐Complex Liposomes

**Figure 3:**
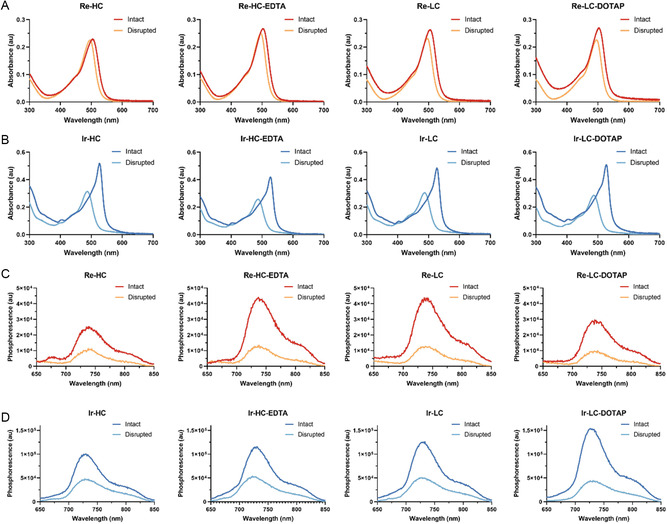
Optical properties of the Re‐ and Ir‐lipid‐based liposomal formulations. A,B) Absorbance spectra of the various Re‐ or Ir‐lipid‐based liposomes in PBS (intact) and in MeOH (disrupted) at a concentration of 10 μm, based on the active lipid (Re‐lipid or Ir‐lipid), respectively. C,D) Emission spectra of the corresponding solutions used for absorbance measurement, with excitation at 495 nm.

The absorbance measurements indicated that intact Re ‐lipid‐based liposomes had a peak absorbance at around 505 nm, which was slightly redshifted compared to their counterpart disrupted samples in MeOH (495 nm), with small variation observed between formulations (**Figure** **3**A). Interestingly, the Ir ‐lipid‐based liposomes exhibited a narrow, redshifted peak (from 487 to 527 nm) with significant increase in absorbance (approximately doubled) compared to their corresponding disrupted samples in MeOH (Figure 3B). The consistent absorbance shift to 527 nm was observed across all Ir‐lipid‐based liposomes, including Ir‐HC, Ir‐LC, Ir‐EDTA, and Ir‐LC‐DOTAP, indicating that Ir‐lipid formed an ordered arrangement in those liposomes. Notably, the color of intact Ir‐lipid‐based liposomes in PBS was visually distinct from its nanostructure‐disrupted counterpart in MeOH while Re‐liposomes showed similar color in both PBS and MeOH condition (Figure S21, Supporting Information).

When excited at 495 nm, Re‐ and Ir‐lipid‐based liposomes emitted a low phosphorescence signal, peaking in the near‐infrared region: 740 nm for Re‐lipid‐based liposome (Figure 3C) and 733 nm for Ir‐lipid‐based liposomes (Figure 3D). Unlike other fluorescence dye‐loaded liposomes that typically show fluorescence quenching in intact nanoparticles and restore fluorescence when nanoparticles are disrupted,^[^ ^21^, ^23^ ^]^ the Re‐ and Ir‐lipid‐based liposomes exhibited even lower phosphorescence when their nanoparticles were disrupted in MeOH. This suggests a more efficient interaction with light in their intact nanoparticle forms. In addition, the phosphorescence generation of Ir‐lipid‐based liposomes was higher than that of Re‐lipid‐based liposomes, exhibiting approximately a 3.5‐fold increase at the same metal complex‐lipid concentration. The generation of emission from Re‐ and Ir‐lipid‐based liposomes showed a temperature‐dependent pattern for both intact and disrupted forms of nanoparticles **(**Figure S22 and S23A,B, Supporting Information). The emission intensity exhibited a decrease with rising temperature, which might be attributed to the increasing dominance of nonradiative processes. Higher temperatures can shorten the duration of phosphorescence and impact its intensity. The phosphorescence emission was sensitive to the presence of oxygen, as evidenced by the stronger emission of Ir‐lipid‐based liposomes in an inert gas (argon) environment compared to under ambient air conditions (Figure S23C, Supporting Information). Furthermore, the phosphorescence signal showed a strong correlation with their concentration in both intact and disrupted forms (Figure S24, Supporting Information).

### 2.5 ROS Generation

**Figure 4:**
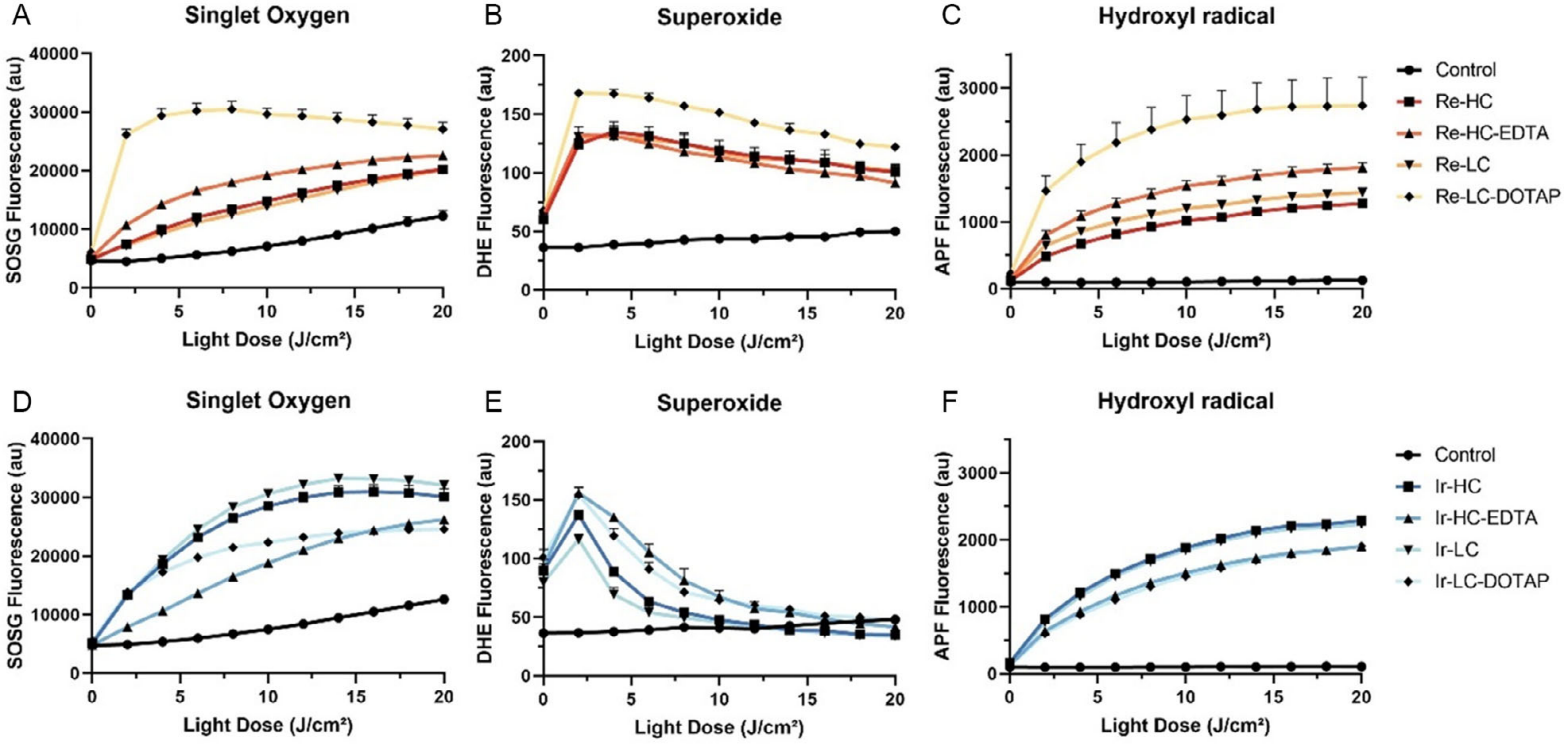
A–F) Light‐dependent generation of specific ROS upon excitation of the Re‐complex‐based (A–C) and Ir‐complex‐based (D–F) liposomes in PBS (10 μm, 450 nm, 70 mW cm^−2^), based on fluorescence assays (*n* = 3).

The fluorescent probes singlet oxygen sensor green (SOSG), dihydroethidium (DHE), and aminophenyl fluorescein (APF) were used to detect the generation of singlet oxygen, superoxide and hydroxyl radical, respectively (**Figure** **4**). All formulations showed light‐dependent singlet oxygen formation, consistent with their original Re‐ and Ir‐complex photosensitizers (Figure 4A,D).^[^ ^19^, ^20^ ^]^ In the case of superoxide (Figure 4B,E), there is an increase in signal at first (at 2 J cm^−1^ ^2^), but then the fluorescence of DHE consistently decreases, possibly due to degradation from other ROS generated in the solution. Nonetheless, there is evidence that this species is also formed. Additionally, the consistent light‐dependent formation of hydroxyl radical (Figure 4C,F) supports the evidence that both Re‐lipid and Ir‐lipid go through type I and type II photodynamic reactions. Re‐LC‐DOTAP stands out from the other Re‐liposomes for having the highest formation of all measured species, but overall, there are only small differences in the ROS generation from formulation to formulation. Exceptionally, the degradation of DHE seems to occur more rapidly in the Ir samples compared to the Re ones.

### 2.6 Photodynamic Efficacy and Selectivity In Vitro

**Figure 5:**
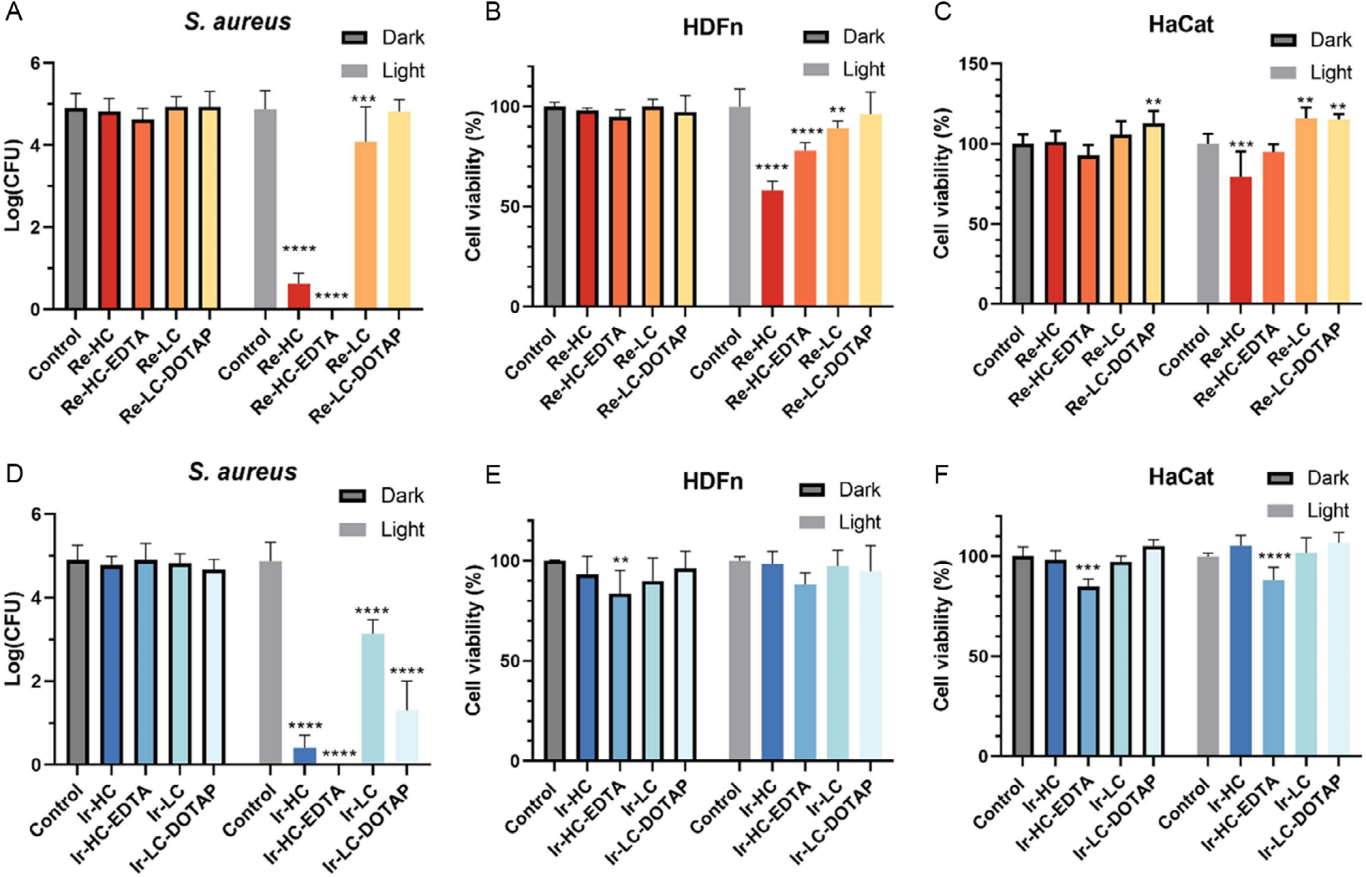
Effect of the liposomal formulations on the viability of *S. aureus* Xen36 ((A) and (D), *n* = 9), HDFn human skin fibroblasts ((B) and (E), *n* = 6), and HaCat human skin keratinocytes ((C) and (F), *n* = 6) with and without light treatment. PDT conditions: 10 μm of active lipid, 15 min of drug–light interval, and 30 J cm^−2^ of 450 nm at 70 mW cm^−2^ for light‐treated groups. Results were compared using a two‐way ANOVA and a post hoc Tukey test, groups significantly different from their respective control are indicated. (*p < 0.05; **p < 0.01; ***p < 0.001; ****p < 0.0001).

The various Re‐ and Ir‐complex‐lipid‐based liposomes were next tested against *S. aureus*, HDFn, and HaCat cells under comparable PDT parameters (**Figure** **5**). The Re‐lipid‐based formulations showed no dark toxicity toward either bacteria or mammalian cells at the tested concentrations. Both Re‐HC and Re‐HC‐EDTA formulations exhibited significant antimicrobial (aPDT) activity against *S. aureus*, resulting in the killing of 4 and 5 logs of bacteria, respectively, under the experimental light treatment conditions (Figure 5A). However, it is important to note that both Re‐HC and Re‐HC‐EDTA formulations also exhibited phototoxicity toward mammalian cell lines (Figure 5B,C). Re‐HC reduced cell viability by 40% in HDFn cells and 20% in HaCat cells, while Re‐HC‐EDTA PDT resulted in ≈25% cell death in HDFn cells. Re‐LC and Re‐LC‐DOTAP demonstrated a higher level of protection toward host cells: Re‐LC showed negligible toxicity to HaCat cells, while exhibited a slight, but not clinically relevant, phototoxicity to HDFn cells, resulting in ≈10% decrease in cell viability under light treatment; Re‐LC‐DOTAP had no significant effect or even slightly increased cell viability for both cell lines under the same light treatment conditions. However, these formulations were found to be less effective against *S. aureus*, with the Re‐LC formulation resulting in only a 1‐log decrease in bacterial viability, which is not considered clinically relevant. Similarly, the Re‐LC‐DOTAP formulation did not show any significant change in bacterial viability. None of the Re‐based formulations had antimicrobial effect against *E. coli* (Figure S25A,B, Supporting Information).

For the Ir‐lipid‐based liposomes, Ir‐HC, Ir‐HC‐EDTA, and Ir‐LC‐DOTAP demonstrated effective antimicrobial activity against *S. aureus*, with reductions of 4, 5, and 3 logs, respectively (Figure 5D). Ir‐LC‐DOTAP was also effective against *E. coli*, but only when treatment conditions were increased to 50 μm of active lipid, 60 min of DLI, and 60 J cm^−2^ (Figure S25C,D, Supporting Information). While Ir‐HC and Ir‐LC‐DOTAP did not show significant adverse effects on HDFn and HaCat cells, Ir‐HC‐EDTA resulted in a partial reduction in cell viability for both cell lines (≈15% decrease, Figure 5E,F). Specifically, a statistically significant dark toxicity of Ir‐HC‐EDTA was observed in both HDFn and HaCat cells, although the reduction in viability did not reach the threshold for clinical relevance (20%). On the other hand, Ir‐LC was safe for mammalian cells but only led to a 2‐log reduction in *S. aureus* viability, which is considered insufficient for an effective antimicrobial treatment. A summary of the results from Figure 5, along with the statistical analysis, is presented in **Table** **2**.

**Table 2:** Cytotoxicity of each liposomal formulation toward each strain (summarized from Figure 5)

Strain	Treatment	Re‐based				Ir‐based			
		Re‐HC	Re‐HC‐EDTA	Re‐LC	Re‐LC‐DOTAP	Ir‐HC	Ir‐HC‐EDTA	Ir‐LC	Ir‐LC‐DOTAP
*S. aureus*	aPDT	Yes (4 logs) ****	Yes (5 logs) ****	Some (1 log) ***	No [ns]	Yes (4 logs) ****	Yes (5 logs) ****	Some (2 logs) ****	Yes (3 logs) ****
	Dark	No [ns]	No [ns]	No [ns]	No [ns]	No [ns]	No [ns]	No [ns]	No [ns]
HDFn	PDT	Yes (40%)****	Yes (25%)****	Some (10%)**	No [ns]	No [ns]	No [ns]	No [ns]	No [ns]
	Dark	No [ns]	No [ns]	No [ns]	No [ns]	No [ns]	Some (15%) **	No [ns]	No [ns]
HaCat	PDT	Yes (20%) ***	No	No (+15%) **	No (+15%) **	No [ns]	Some (15%) ****	No [ns]	No [ns]
	Dark	No [ns]	No [ns]	No [ns]	No (+15%) **	No [ns]	Some (15%) ***	No [ns]	No [ns]

a) Results were compared using a two‐way ANOVA and a post hoc Tukey test. Groups significantly different from their respective control are indicated (*p < 0.05; **p < 0.01; ***p < 0.001; ****p < 0.0001). Results listed as “some” refer to statistically significant decrease when compared to the respective control, but that does not meet the criteria for clinically relevant toxicity (3 logs for bacteria and 20% for mammalian cells).

## 3 Discussion

### 3.1 Photodynamic Activity of Re‐ and Ir‐Lipid

Both Re and Ir lipids, as well as their corresponding liposomes, exhibit large Stokes shifts, characteristic of the phosphorescence emission of organometallic complexes (Figure 1C and 3C,D).^[^ ^24^ ^]^ The Re‐ and Ir‐lipid‐based liposomes demonstrated a more efficient interaction with light in their intact nanoparticle forms compared to the free Re‐ or Ir‐lipid, as evidenced by their higher phosphorescence when the nanoparticles were intact rather than disrupted. Furthermore, the Ir‐lipid‐based formulations exhibited higher extinction coefficients (twofold) and greater phosphorescence generation (>threefold) compared to the Re‐lipid‐based liposomes (Figure 3) at the same metal complex‐lipid concentration.

The Ir‐lipid‐based liposomes demonstrated better selectivity for aPDT in vitro (Figure 4 and Table 2) under the experimental conditions. Three out of the four Ir‐lipid‐based formulations, Ir‐HC, Ir‐HC‐EDTA, and Ir‐LC‐DOTAP, achieved a reduction of over 3 logs of *S. aureus*, while only two of the Re‐lipid‐based formulations (Re‐HC and Re‐HC‐EDTA) achieved the same level of reduction. Additionally, only Ir‐HC‐EDTA exhibited toxicity toward host cells, which remained below the 20% threshold, while Re‐HC and Re‐HC‐EDTA exhibited significant host cell toxicity.

The wide absorbance bands from 400 to 550 nm for both Re‐ and Ir‐lipid‐based liposomes allow for excitation of all the samples in the blue–green range. It is important to note that a 450 nm light source was used for the experimental light treatment, which is not the peak absorbance wavelength for either of the liposomes. Despite the use of a nonideal wavelength, all formulations showed light‐dependent ROS generation and most also presented bacterial inactivation (Figure 4 and 5A,D), indicating that the chosen wavelength was still effective in activating the Re‐ and Ir‐lipid‐based nanoparticles and achieving PDT efficacy. The choice of wavelength for aPDT treatment takes into account factors beyond peak absorbance. Blue LEDs, for example, are more powerful and efficient,^[^ ^25^ ^]^ while green light can penetrate deeper into biological tissue.^[^ ^26^ ^]^ Therefore, the activation wavelength for Re‐ and Ir‐lipid‐based liposomes can be modulated based on the specific application.

### 3.2 Effect of Formulation Components (Cholesterol, PEG‐Lipid, Base Lipid Length)

The initial base formulations of the metal‐complex liposomes, Re‐HC and Ir‐HC, were designed with a composition of 40% cholesterol and 5% PEG‐lipid (Table 1). These formulations were inspired by the successful use of improved porphyrin‐based liposomes in cancer PDT.^[^ ^22^ ^]^ Cholesterol was incorporated in the liposomal formulations as a stabilizer, providing increased packing of phospholipids and enhancing resistance to shear stress.^[^ ^27^ ^]^ Additionally, higher cholesterol content has been shown to enhance the penetration of liposomes into the skin.^[^ ^28^ ^]^ On the other hand, PEG‐lipid is commonly incorporated into liposomal formulations to enhance their stability and prolong the circulation time of liposomes in the bloodstream. However, the effect of PEG‐lipid on cellular uptake can vary depending on factors such as surface density, and the specific cell type being targeted.^[^ ^29^ ^]^ In the context of antimicrobial applications, the goal was to design a formulation that would enhance the uptake of liposomes by the target pathogen while minimizing uptake by host cells to avoid tissue damage and impairments in wound healing. To investigate this, an alternative base formulation was prepared by reducing the amount of cholesterol and PEG‐lipid and replacing DSPC with the less rigid DPPC lipid. This resulted in the formulation of Re‐LC and Ir‐LC liposomes (Table 1). It was interesting to find that, despite generating similar amounts of ROS, Re‐HC and Ir‐HC formulations exhibited significant phototoxicity against *S. aureus*, while Re‐LC and Ir‐LC formulations did not show the same level of efficacy (Figure 4 and 5), which suggests that one or a combination of the varying elements in the formulations may be crucial for the effectiveness of aPDT under the tested conditions. However, Re‐HC also demonstrated toxicity toward host cells, which was significantly reduced in the Re‐LC formulation (Figure 5B,C). Despite the change in formulation components, the selectivity of Re‐lipid liposomes for aPDT was not improved, as the increase in efficacy was accompanied by increased toxicity to host cells. On the other hand, the Ir‐lipid‐based formulations, including Ir‐HC and Ir‐LC, did not show any toxicity toward host cells under the tested conditions (Figure 5E,F). Moreover, Ir‐HC was effective against the pathogen (4 logs, see Figure 5D and Table 2), fulfilling the in vitro selectivity requirement for aPDT.

### 3.3 Effect of Incorporation of EDTA

Unlike EDTA, which is often included in formulations as a metal chelating agent, the incorporation of EDTA‐lipid into liposomal nanoparticles enables the fluidization of the cell membrane in a detergent‐like manner, promoting intramembrane uptake of nanoparticles without affecting cell membrane integrity. This enhancement in membrane permeability has been shown to improve the PDT efficacy of porphyrin liposomes.^[^ ^22^ ^]^ By engineering EDTA‐lipid into Re‐ and Ir‐lipid‐based liposomes, we did not observe a dramatic increase in singles oxygen generation (Figure 4), but there was a prompt enhancement in bacterial killing for both Re‐HC‐EDTA and Ir‐HC‐EDTA, leading to the complete inactivation of *S. aureus* (Figure 5A,D). We also investigated the potential side effects of EDTA‐lipid on host cells and found mild changes to the ‐HC counterparts: Re‐HC‐EDTA exhibited lower side effects on HDFn cells compared to the Re‐HC formulation without EDTA‐lipid (Figure 5B, 25% vs 40%), and it showed no effect on HaCat cells (Figure 5C); on the other hand, Ir‐HC‐EDTA slightly decreased the viability of both cell lines, but to a degree that is not considered clinically relevant.

### 3.4 Effect of the Incorporation of DOTAP

DOTAP, a positively charged amphiphilic lipid, has been extensively used in liposomal formulations for drug delivery, particularly in gene therapy.^[^ ^30^ ^]^ As the bacterial cell wall tends to have a negative charge, DOTAP has been explored as a component of antimicrobial liposomes to facilitate the interaction between the nanoparticle and its target through electrostatic attraction.^[^ ^31^, ^32^ ^]^ In the case of the Re‐ and Ir‐lipid‐based formulations, the inclusion of DOTAP resulted in a shift in the surface charge of the liposomes from negative to positive. Re‐LC‐DOTAP and Ir‐LC‐DOTAP exhibited Zeta potentials of 0.99 and 1.65 mV, respectively, while Re‐LC and Ir‐LC had Zeta potentials of −4.10 and −3.17 mV, respectively (Table 1). It was anticipated that these positively charged nanoparticles would exhibit enhanced interaction with negatively charged bacterial cells, thereby increasing their antimicrobial potency. The results showed that Ir‐LC‐DOTAP demonstrated a statistically significant enhancement in antimicrobial activity, achieving clinically relevant efficacy under the tested conditions, while Ir‐LC without DOTAP only showed partial aPDT efficacy (Figure 5D and S25D, Supporting Information). However, this was not the case for the Re‐lipid‐based formulations, as neither Re‐LC nor Re‐LC‐DOTAP exhibited any aPDT response against *S. aureus* nor *E. coli* under the experimental conditions (Figure 5A, and S25A,B, Supporting Information), despite the significant increase in reaction rates and absolute amounts of singlet oxygen, superoxide, and hydroxyl radical generated by Re‐LC‐DOTAP (Figure 4A–C). This lack of response may suggest a poorer membrane interaction of this Re‐lipid formulation compared to the Ir‐lipid one. TEM images in Figure 2 displayed a varied distribution of sizes for the DOTAP‐containing liposomes, indicating some heterogeneity in particle size. DLS measurements further revealed an increase in particle size for both Re‐LC‐DOTAP (from 109.8 to 171.9 nm) and Ir‐LC‐DOTAP (from 157.2 to 179.9 nm) after 2 days of storage at 4 °C postpreparation. These observations suggest that Re‐LC‐DOTAP and Ir‐LC‐DOTAP liposomes may coalesce and aggregate over time under 4 °C storage.

### 3.5 Considerations on the aPDT of Chronic Skin Wounds

Due to the complex nature of chronic wounds, including the impaired tissue regeneration and healing ability of the body, it is crucial to consider drug selectivity for effective treatment. However, many previous studies on aPDT for chronic wounds have overlooked this aspect. We propose that the impact of the treatment on healthy host cells should be given equal importance to bacterial killing. Considering that topical application is often preferred for treating chronic wound infections due to its avoidance of first‐pass metabolism and many systemic side effects,^[^ ^3^ ^]^ the potential toxicity of the treatment becomes particularly significant in the localized skin microenvironment. Therefore, we selected keratinocytes and skin fibroblasts as controls to evaluate the selectivity of the proposed aPDT treatment in vitro.

Among the eight liposomal formulations described in Table 1, most showed efficacy against *S. aureus* in suspension (Re‐HC, Re‐HC‐EDTA, Ir‐HC, Ir‐HC‐EDTA, and Ir‐LC‐DOTAP). Three‐log inactivation using the aPDT parameters of this study (10 μm, 15 min DLI, 30 J cm^−2^) is satisfactory when compared to many other proposed previously protocols for *S. aureus* in wound infections. For example, Pourhajibagher et al. achieved significant (1 log) inactivation of using 40 μm of indocyanine green, 5 min DLI and 31.2 J cm^−2^;^[^ ^33^ ^]^ Mirzahosseinipour et al. achieved 3 log inactivation using a curcumin‐based nanoparticle at 135 μm, 30 min DLI and 20 J cm^−2^;^[^ ^34^ ^]^ and Zhao et al. obtained <2 log inhibition with 15.6 μm of the porphyrin derivative PPIX‐MED, 30 min DLI and 6 J cm^−2^.^[^ ^35^ ^]^

A similar relationship between formulation composition and aPDT efficacy was observed for an *S. aureus* biofilm model (Figure S26, Supporting Information), although the treatment conditions were not strong enough to produce significant results. Biofilms are much more resistant to treatment than bacteria in suspension, and it was expected that this protocol would not be sufficient. As an example, the super‐potent porphyrin nanoemulsion presented by Buzzá et al significantly killed *S. aureus* in suspension with concentrations as little as 50 nm, but results were only seen in biofilm with at least 10 μm.^[^ ^36^ ^]^ Similarly, the Re‐ and Ir‐liposomes were only effective against *E. coli* at higher concentration, DLI and light dose (Figure S25B,D, Supporting Information).

Unfortunately, three of the most effective formulations (Re‐HC, Re‐HC‐EDTA, and Ir‐HC‐EDTA) already demonstrated some level of phototoxicity toward host cells even with the original aPDT protocol. Thus, only two formulations, namely, Ir‐HC and Ir‐LC‐DOTAP, met the proposed selectivity criteria under the tested conditions. Ir‐LC‐DOTAP exhibited poor colloidal stability, posing a challenge for its further development in clinical application, so Ir‐HC was the only formulation that demonstrated ideal characteristics for chronic wound aPDT, being effective, selective, and stable under the tested conditions.

## 4 Conclusion

Ir‐ and Re‐complex lipid‐based liposomal formulations demonstrate potential as aPDT agents for treating infected skin wounds. We investigated the modification of the liposome composition to optimize their aPDT efficacy and selectivity against bacteria while minimizing toxicity to host cells to prevent tissue damage and impairments in wound healing, and have found that the structural composition of the nanoparticles significantly impacts their response. Based on our findings, we draw the following conclusions: Ir‐lipid‐based liposomes exhibited superior aPDT efficacy against *S. aureus* compared to Re‐lipid‐based liposomes while generally also demonstrating better tolerability toward host cells. Liposomes with higher cholesterol and PEG‐lipid content exhibited increased potency against *S. aureus*, highlighting the importance of these components in enhancing antimicrobial activity. The incorporation of EDTA‐lipid significantly enhanced aPDT efficacy, but it also increased toxicity toward host cells. Therefore, further optimization of treatment parameters is necessary to balance antimicrobial potency and host cell compatibility for this modification. The incorporation of cationic DOTAP altered the surface charge of the nanoparticles to positive, potentially improving interaction with bacterial cell walls and enhancing efficacy. However, this modification negatively impacted the stability of the formulations. Overall, among the tested formulations, Ir‐HC liposomes demonstrated excellent efficacy, selectivity, and stability, making them the most promising candidate for aPDT in the treatment of wound infections under the experimental conditions.

## 5 Experimental Section

5.1

5.1.1

##### General Materials and Methods

Cholesterol, 1‐hexadecanoyl‐*sn*‐glycero‐3‐phosphocholine (16:0 Lyso PC), 1,2‐distearoyl‐*sn*‐glycero‐3‐phosphoethanolamine‐*N*‐[methoxy(polyethylene glycol)‐2000] (DSPE‐mPEG2000), 1,2‐dipalmitoyl‐*sn*‐glycero‐3‐phosphocholine (DPPC), 1,2‐distearoyl‐*sn*‐glycero‐3‐phosphocholine (DSPC), and 1,2‐dioleoyl‐3‐trimethylammonium‐propane (chloride salt) (DOTAP) were purchased from Avanti Polar Lipids (USA). All other solvents and reagents were of reagent grade and obtained from Sigma–Aldrich (USA). Water was purified with Milli‐Q Plus 185 water purification system (Millipore, Bedford, MA, USA). The ESI‐MS was measured using a Waters Acuity uPLC. The uPLC‐MS system was assembled with a Waters 2695 controller, a 2996 photodiode array detector, a Waters triple quadrupole mass detector, and a BEH C18 1.7 μm column (Waters Canada, Ontario, Canada). The uPLC flow method used the following parameters: Solvent A) 0.1% TFA and Solvent B) acetonitrile; column temperature: 60 °C; flow rate: 0.6 mL min^−1^; gradient from 60% A + 40% B to 0% A + 100% B in 3 min, kept at 100% B for 1 min, followed by a sharp change back to 60% A + 40% B. The NMR spectra were recorded on a Bruker Ultrashield 400 Plus NMR spectrometer (^1^H NMR 400.18 MHz) with TMS as internal standard.

##### Synthesis and Characterization of Re Dipyrrinate Mercaptopropionic Acid (Compound **4**) and Ir Dipyrrinate Mercaptopropionic Acid (Compound **6**)

3‐Mercaptopropionic acid (2.6 equiv.) was dissolved in DMF under an inert atmosphere. Diethanolamine (DEA) was added and stirred for 5 min. To this reaction mixture, either Re dipyrrinate (**3**)^[^ ^19^ ^]^ or Ir dipyrrinate (**5**)^[^ ^20^ ^]^ (1.0 equiv.) was added and further stirred at room temperature for 4 h. The progress of reactions was monitored using TLC, and the solvent was subsequently removed under reduced pressure. The resulting mixtures were redissolved in dichloromethane and washed three times with a saturated solution of ammonium chloride (25 mL each), and then dried over sodium sulfate. Upon removing the solvent under reduced pressure, the desired pure compound was obtained through multiple washes with hexane and pentane. Finally, orange solid products were obtained.

##### Compound (**4**)

The reaction was set up with 3‐mercaptopropionic acid (262 mg, 205 μL, 2.47 mmol, 2.6 equiv.), DMF (5 mL), DEA (2 mL), Re dipyrrinate (**3**) (800 mg, 0.95 mmol, 1 equiv.), resulting in 800 mg compound (**4**) (orange solid with yield of 90%). ^1^H NMR (400 MHz, CDCl_3_, *δ* ppm): 7.48 (d, *J* = 1.2 Hz, 2H, α‐pyrrolic‐H, *a*), 7.33‐7.31 (m, 3H, Ar–H, *f*), 7.27‐7.26 (m, 1H, Ar–H, *e*), 7.25‐7.22 (m, 5H, Ar–H, *e*), 7.13–7.08 (m, 6H, Ar–H, *d*), 6.43 (d, *J* = 4.4 Hz, 2H, β‐pyrrolic‐H, *c*), 6.20 (dd, *J* = 1.6, 2.8 Hz, 2H, β‐pyrrolic‐H, *b*), 3.28 (t, *J* = 7.2 Hz, 2H, —S—CH_2_—C—, *g*), 2.78 (t, *J* = 7.2 Hz, 2H, —C—CH_2_—COOH, *h*) (Figure S1, Supporting Information); ^13^C NMR (100.6 MHz, CDCl_3_, *δ* ppm): 196.4, 176.3, 155.6, 155.6, 135.1, 133.5, 133.4, 132.1, 130.7, 130.3, 130.0, 129.9, 129.7, 128.4, 128.3, 120.0, 119.9, 118.4,114.2, 34.8, 29.3 (Figure S2, Supporting Information); ^31^P NMR (161.9 MHz, CDCl_3_, *δ* ppm): 16.00 (s, 1P, *P*Ph_3_) (Figure S3, Supporting Information); ^19^F NMR (376.49 MHz, CDCl_3_, *δ* ppm): −133.02 (dd, *J* = 11.29, 15.05 Hz, 1F, Ar–F_ortho_), −133.61 (dd, *J* = 11.29, 15.05 Hz, 1F, Ar–F_ortho_), −138.47 (dd, *J* = 11.29, 15.05 Hz, 1F, Ar–F_meta_), −139.92 (dd, *J* = 11.29, 15.05 Hz, 1F, Ar–F_meta_) (Figure S4, Supporting Information); and ESI‐MS: C_39_H_27_F_4_N_2_O_5_PReS^+^ [M + H]^+^: calcd *m*/*z* 929.0872, found *m*/*z* 929.0 (Figure S5, Supporting Information).

##### Compound (**6**)

The reaction was set up with 3‐mercaptopropionic acid (528 mg, 433 μL, 4.97 mmol, 2.6 equiv.), DMF (6 mL), DEA (4 mL), Ir dipyrrinate (**5**) (1.5 g, 1.914 mmol, 1 equiv.), resulting in 1.5 g compound (**6**) with yield of 88%; ^1^H NMR (400 MHz, CDCl_3_, *δ* ppm): 7.92 (d, *J* = 4.8 Hz, 2H, Ar–H, *k*), 7.82 (d, *J* = 8 Hz, 2H, Ar–H, *h*), 7.64–7.60 (m, 4H, Ar–H, *i*, *g*), 6.94‐6.91 (m, 4H, Ar–H, *j*, *f*), 6.85–6.79 (m, 4H, Ar–H, *e* and α‐pyrrolic‐H, a), 6.47 (d, *J* = 4 Hz, 2H, *β*‐pyrrolic‐H, *c)*, 6.39 (dd, *J* = 1.2, 6.4 Hz, 2H, Ar–H, d), 6.26 (dd, *J* = 1.2, 3.2 Hz, 2H, β‐pyrrolic‐H, *b*), 3.26 (t, *J* = 7.2 Hz, 2H, —S—CH_2_—C—, *l*), 2.75 (t, *J* = 7.2 Hz, 2H, —C—CH_2_—COOH, *m*) (Figure S6, Supporting Information); ^13^C NMR (100.6 MHz, DMSO, *δ* ppm): 173.0, 168.2, 155.6, 153.4, 149.2, 145.7, 145.1, 137.8, 133.2, 131.9, 131.6, 130.8, 129.8, 124.8, 123.2, 121.4, 119.8, 119.0, 118.6, 114.3, 114.1, 35.3, 30.1 (Figure S7, Supporting Information). ^19^F NMR (376.49 MHz, DMSO, *δ* ppm): −134.03 (dd, *J* = 11.29, 15.05 Hz, 2F, Ar–F_ortho_), −141.84 (dd, *J* = 11.29, 15.05 Hz, 2F, Ar–F_meta_) (Figure S8, Supporting Information). ESI‐MS: C_40_H_27_F_4_IrN_4_O_2_S^+^ [M]^+^: calcd *m*/*z* 896.1420, found m/z 896.0 (Figure S9, Supporting Information).

##### Synthesis and Characterization of Re‐ and Ir‐Lipid (Compounds **1** and **2**): Re‐Lipid (**1**)

Compound (**4**) (400 mg, 0.43 mmol, 1 equiv.), 1‐palmitoyl‐2‐hydroxy‐*sn*‐glycero‐3‐phosphocholine (214 mg, 0.43 mmol, 1 equiv.), DIPEA (33 mg, 45 μL, 0.26 mmol, 0.6 equiv.), DMAP (26 mg, 0.21 mmol, 0.5 equiv.), and HBTU (408 mg, 1.07 mmol, 2.5 equiv.) were dissolved in anhydrous CHCl_3_ (15 mL) under an argon inert atmosphere and stirred at room temperature for 5 days. After removing the solvent, the residue was directly added onto a flash chromatography silica gel column and purified using an initial gradient of 100% chloroform and a second gradient of MeOH:CHCl_3_ (0–25%) to remove impurities. The pure product was then collected by flashing with CHCl_3_:MeOH:water (47–43:2.5–6.5:0.5) to obtain an orange solid with a yield of 110 mg (18%). ^1^H NMR (400 MHz, CDCl_3_, *δ* ppm): 7.45 (d, *J* = 0.8 Hz, 2H, α‐pyrrolic‐H, *a*), 7.32–7.28 (m, 3H, Ar–H, *f*), 7.24–7.20 (m, 6H, Ar–H, *e*), 7.11–7.06 (m, 6H, Ar–H, *d*), 6.43 (d, *J* = 4 Hz, 2H, β‐pyrrolic‐H, *c*), 6.20 (dd, *J* = 1.2, 3.2Hz, 2H, β‐pyrrolic‐H, *b*), 5.29 (s, 1H, —*O*—CH—(CH_2_)_2_—, *n*), 4.46–4.18 (m, 3H, —CH_2_—CH_2_—*O*—*, p* and –CH—CH—*O*—, *o*), 4.17‐4.11 (m, 3H, —CH–CH—O—, *o* and —CH—CH_2_—*O*—, *m*), 3.89 (s, 2H, —N—CH_2_—CH_2_—, *q*), 3.36 (s, 9H, —N—CH_3_, *r*), 3.26–3.25 (m, 2H, —S—*C*H_2_—CH_2_—, *g*), 2.74‐2.71 (m, 2H, —CH_2_—CH_2_—COO‐, *h*), 2.30 (t, *J* = 7.6 Hz, —OOC—CH_2_—C—, *l*), 1.56–1.55 (m, 2H, —C—CH_2_—C—, *k*), 1.24–1.22 (m, 24H, —C—(CH_2_)_12_—C—, *j*), 0.85 (t, *J* = 6.8 Hz, 3H, —C—CH_3_, *i*) (Figure S10, Supporting Information); ^13^C NMR (100.6 MHz, CDCl_3_, *δ* ppm): 196.2, 173.6, 170.7, 157.3, 155.6, 139.3, 135.1, 133.5, 133.4, 130.6, 130.2, 129.9, 129.8, 128.3, 120.0, 118.2, 114.5, 106.7, 70.8, 66.1, 64.5, 62.3, 60.3, 54.5, 40.1, 34.9, 34.0, 31.9, 29.7, 29.7, 29.6, 29.4, 29.2, 24.9, 22.7, 14.1 (Figure S11, Supporting Information); ^31^P NMR (161.9 MHz, CDCl_3_, *δ* ppm): 15.99 (s, 1P, PPh_3_), −2.22 (s, 1P, phosphate group) (Figure S12, Supporting Information); ^19^F NMR (376.49 MHz, CDCl_3_, *δ* ppm): −133.25 (dd, *J* = 11.29, 15.05 Hz, 1F, Ar–F_ortho_), −133.50 (dd, *J* = 11.29, 15.05 Hz, 1F, Ar–F_ortho_), −138.89 (dd, *J* = 11.29, 15.05 Hz, 1F, Ar–F_meta_), −139.96 (dd, *J* = 11.29, 15.05 Hz, 1F, Ar–F_meta_) (Figure S13, Supporting Information). ESI‐MS C_63_H_75_F_4_N_3_O_11_P_2_ReS^+^ [M + H]^+^: calcd *m/z* 1406.4091, found *m/z* 1406.20 (Figure S14, Supporting Information).

##### Synthesis and Characterization of Re‐ and Ir‐Lipid (Compounds **1** and **2**): Ir‐Lipid (**2**)

Compound (**6**) (500 mg, 0.55 mmol, 1 equiv.), 1‐palmitoyl‐2‐hydroxy‐*sn*‐glycero‐3‐phosphocholine (277 mg, 0.55 mmol, 1 equiv.), *N*,*N*‐diisopropylethylamine (87 mg, 116 μL, 0.66 mmol, 1.2 equiv.), 4‐dimethylaminopyridine (55 mg, 0.45 mmol, 0.8 equiv.), and HBTU (635 mg, 1.67 mmol, 3 equiv.), CHCl_3_ (15 mL) stirred at room temperature for 4 days. After 4 days, the residue was directly added onto a flash chromatography silica gel column, and purified using an initial gradient of 100% chloroform, second gradient with MeOH:CHCl_3_ (0–25%) to remove out impurities, the pure product collected using CHCl_3_:MeOH:water (47–42:2.5–6.5:0.5). An orange solid was obtained. Yield: (151 mg, 20%); ^1^H NMR (400 MHz, CDCl_3_, δ ppm): 7.87 (d, *J* = 5.6 Hz, 2H, Ar–H, k), 7.75 (d, *J* = 8 Hz, 2H, Ar–H, *h*), 7.58–7.50 (m, 4H, Ar–H, *i*, *g*), 6.92–6.88 (m, 2H, Ar–H, *j*), 6.85–6.77 (m, 6H, Ar–H, *f*, *e* and α‐pyrrolic‐H, *a*), 6.46 (d, *J* = 4 Hz, 2H, *β*‐pyrrolic‐H, *c*), 6.37 (d, *J* = 7.2 Hz, 2H, Ar–H, *d*), 6.21 (d, *J* = 4 Hz, 2H, *β*‐pyrrolic‐H, *b*), 5.25 (s, 1H, —*O*—CH—(CH_2_)_2_—, *s*), 4.44‐4.33 (m, 3H, —CH_2_—CH_2_—O—*, u* and –CH—CH—*O*—*, t*), 4.17–4.08 (m, 3H, —CH—CH—O—*, t* and –CH—CH_2_—O—*, r*), 3.86 (s, 2H, —N—CH_2_—CH_2_—, *v*), 3.31 (s, 9H, —N—CH_3_, *w*), 3.25–3.22 (m, 2H, —S—CH_2_—CH_2_—, *l*), 2.74–2.70 (m, 2H, —CH_2_—CH_2_—COO—, *m*), 2.27 (t, *J* = 7.6 Hz, —OOC—CH_2_—C‐, *q*), 1.54–1.51 (m, 2H, —C—CH_2_—C—, *p*), 1.23–1.21 (m, 24H,—C—(CH_2_)_12_—C—, *o*), 0.86 (t, *J* = 6.8 Hz, 3H, —CH_2_—CH_3_, *n*) (Figure S15, Supporting Information); ^13^C NMR (100.6 MHz, CDCl_3_, *δ* ppm): 173.6, 170.7, 168.5, 155.6, 153.8, 149.6, 144.5, 136.2, 132.9, 132.2, 131.1, 129.6, 129.5, 126.9, 123.9, 121.9, 120.9, 118.6, 118.4, 111.0, 106.7, 65.9, 62.2, 60.3, 54.4, 40.1, 34.0, 31.9, 29.7, 29.6, 29.5, 29.3, 29.2, 24.8, 22.7, 14.2 (Figure S16, Supporting Information); ^31^P NMR (161.9 MHz, CDCl_3_, *δ* ppm): −2.27 (s, 1P, Phosphate group) (Figure S17, Supporting Information); ^19^F NMR (376.49 MHz, CDCl_3_, *δ* ppm): −133.65 to −133.75 (m, 2F, Ar–F_ortho_), −139.68 to −139.80 (m, 2F, Ar–F_meta_) (Figure S18, Supporting Information); ESI–MS: C_64_H_76_F_4_IrN_5_O_8_PS ^+^ [M + H]^+^: calcd *m/z* 1374.4718, found *m/z* 1374.90 (Figure S19, Supporting Information).

##### Synthesis of Liposome Formulations

Various nanoparticles containing Re‐ and Ir‐lipid were synthesized following a previously reported freeze and thaw method for liposomes.^[^ ^21^ ^]^ Briefly, the lipid components consisting of Re‐ or Ir‐lipids, cholesterol, DSPE‐mPEG2000, and other amphiphilic phospholipids at different molar ratios (Table 1) were dissolved and well mixed in chloroform. The mixtures were dried under a gentle stream of nitrogen gas. Then, the dried lipid films were rehydrated with phosphate buffered saline (PBS) (150 mm, pH 7.5) at the concentration of 3 mg mL^−1^, underwent the freeze‐and thaw process seven times, and then were extruded through a polycarbonate membrane (pore size = 100 nm) seven times to obtain various liposome formulations.

##### Nanoparticle Characterization

DLS measurements were performed in PBS to determine the size and polydispersity index of the prepared liposome formulations using a ZS90 Nanosizer system (Malvern Instruments). Zeta potentials of formulations in double distilled water (ddH_2_O) were measured as well. The morphologies of the various formulations were analyzed using Hitachi HT7800 TEM (Hedwig, Hitachi High Technologies). To prepare the TEM samples, each formulation was deposited on carbon–copper‐coated grids for 2 min, followed by three washes with ddH_2_O. Finally, the samples were negatively stained with 2% uranyl acetate and air‐dried before the TEM imaging. The absorbance and fluorescence spectra of intact (in PBS) and disrupted (in MeOH) nanoparticles were measured using the UV/vis spectrophotometer Cary 50 (Agilent, Mississauga, ON, USA) and the Fluoromax‐4 spectrofluorometer (Horiba Jobin Yvon, USA) with an excitation wavelength of 495 nm and an emission range of 650‐850 nm, using a 5 nm slit width.

##### ROS Quantification

ROS generation was detected using fluorescent probes, namely, SOSG (Invitrogen) for singlet oxygen, DHE (Abcam) for superoxide, and the APF (Invitrogen) for hydroxyl radical. Briefly, samples containing each formulation in PBS at a concentration of 10 μm of the active lipids were added to 24‐well plates and combined with SOSG, DHE, or APF (also at 10 μm), and then exposed to incremental treatments of 450 nm light (2 J cm^−1^ ^2^ at a time, 70 mW cm^−2^) using a custom‐made 24‐LED array (*n* = 3). The fluorescence of the oxidized probes (SOSG 504/525 nm, DHE 515/610 nm, and APF 490/515 nm) was measured using a plate reader.

##### In Vitro Culture Conditions

*S. aureus* Xen36 (PerkinElmer) and *E. coli* DH5α with the pZE27GFP plasmid added (Addgene) were grown from frozen stocks onto brain–heart infusion (BHI) agar overnight at 37 °C. Three to five isolated colonies were transferred to BHI broth containing 200 μg mL^−1^ of kanamycin and grown under agitation (220 rpm) at 37 °C for about 18 h. Then, 30 μL of this bacterial suspension were transferred to a fresh 3 mL aliquot of the same broth and grown to log‐phase (about 2 h) in the same conditions. The optical density was determined by measuring the absorbance at 600 nm and then the suspensions were centrifuged (at 1300 g for 10 min) and resuspended in PBS to an adjusted concentration of 10^7^ colony‐forming units (CFU) mL^−1^.

HaCat human keratinocytes (RRID: CVCL_0038, obtained from the Eleftherios Diamandis Lab in Mt. Sinai Hospital) and HDFn primary human dermal fibroblasts (PCS‐201‐010, purchased from ATCC) were grown from frozen stocks in Dulbecco's modified Eagle medium supplemented with 10% fetal bovine serum (FBS) at 37 °C and 5% CO_2_. They were seeded onto 24‐well plates at a density of 1 × 10^5^ cells per well 18‐24 h prior to each experiment.

##### In Vitro aPDT and PDT

For the antimicrobial assays, each liposomal formulation was diluted in PBS to a concentration of 20 μm then combined with the bacterial suspension in 24‐well plates in a proportion of 1:1, yielding final concentrations of 10 μm of active lipid and 5 × 10^6^ CFU mL^−1^ of *S. aureus* in a total volume of 500 μL. For the mammalian cell toxicity assays, the liposomal formulations were diluted Fluorobrite DMEM supplemented with 10% FBS and 4 mm l‐glutamine to a final concentration of 10 μm and then 500 μL were added to each well where the cells were attached, after removing the old medium.

The bacterial and mammalian cell 24‐well plates were placed back into their respective incubators for 15 min (drug‐light interval or DLI), and then the light‐exposed (aPDT or PDT) groups were treated with 30 J cm^−1^ ^2^ of light at 450 nm with an irradiance of 70 mW cm^−1^ ^2^, using a custom‐made light box composed of 24 individual LED sources, arranged in a way to overlap correctly with 24‐well plates. During that time, the nonlight‐exposed (dark) groups were kept in the incubator.

The bacterial samples were serially diluted in PBS immediately after the light treatment and seeded onto BHI agar plates, and after overnight incubation at 37 °C, the CFUs were counted.

The HDFn and HaCat cells were incubated for an additional 18–24 h after the light treatment, and then underwent the Alamar Blue viability assay. Briefly, resazurin (Alamar Blue) was diluted in supplemented Fluorobrite DMEM to a concentration of 50 μg mL^−1^ and added to the wells replacing the old medium. After 2 or 3 h of incubation (HaCat and HDFn, respectively), resofurin fluorescence was measured using a plate reader (excitation: 540 nm, emission: 590 nm) and groups were compared to nontreated (100%) and blank (0%) controls.

##### Statistical Analysis

Data were processed and plotted using GraphPad Prism 8.0. When applicable, statistical analysis was performed using the same software. Results were deemed statistically significant when *p* < 0.05, and presented according to *p*‐value (*p < 0.05; **p < 0.01; ***p < 0.001; ****p < 0.0001). Nanoparticle size and Zeta potential results are representative from three measurements. ROS generation results are presented as mean ± standard deviation from *n* = 3. The *S. aureus* aPDT experiments were performed in triplicate, on three separate occasions (total *n* = 9), and CFU count results were log‐transformed to enable a parametric two‐way analysis of variance (ANOVA) test. A post hoc multiple comparisons Dunnett's test was then performed to compare different treatments. PDT experiments in mammalian cell lines were performed in triplicate on two separate occasions (total *n* = 6). The fluorescence measurements were transformed so that the controls would be 100% and the no‐cell wells would be 0%, and then the two‐way ANOVA and post hoc Dunnett's tests were performed.

## Conflict of Interest

The authors declare no conflict of interest.

## Supporting information

Supplementary Material

## Data Availability

The data that support the findings of this study are available from the corresponding author upon reasonable request.

## References

[1] K. S. Kaye , L. A. Petty , A. F. Shorr , M. D. Zilberberg , Clin. Infect. Dis. 2019, 68, S193. 30957165 10.1093/cid/ciz002 PMC6452002

[2] M. Sisay , T. Worku , D. Edessa , BMC Pharmacol. Toxicol. 2019, 20, 35. 31146791 10.1186/s40360-019-0315-9 PMC6543595

[3] L. F. S. Gushiken , F. P. Beserra , J. K. Bastos , C. J. Jackson , C. H. Pellizzon , Life 2021, 11, 665. 34357037 10.3390/life11070665 PMC8307436

[4] J. Hurlow , P. G. Bowler , J. Wound Care 2022, 31, 436. 35579319 10.12968/jowc.2022.31.5.436

[5] M. Wainwright , T. Maisch , S. Nonell , K. Plaetzer , A. Almeida , P. George , G. P. Tegos , M. R. Hamblin , U. R. Llull , Lancet Infect. Dis. 2017, 17, 49. 10.1016/S1473-3099(16)30268-7 PMC5280084 27884621

[6] J. Oyama , Á. C. F. H. Ramos-Milaré , D. S. S. L. Lera-Nonose , V. Nesi-Reis , I. G. Demarchi , S. M. A. Aristides , J. J. V. Teixeira , T. G. V. Silveira , M. V. C. Lonardoni , Photodiagnosis Photodyn. Ther. 2020, 30, 101682. 32032780 10.1016/j.pdpdt.2020.101682

[7] Y. Sun , R. Ogawa , B. H. Xiao , Y. X. Feng , Y. Wu , L. H. Chen , X. H. Gao , H. D. Chen , Int. Wound J. 2020, 17, 285. 31724831 10.1111/iwj.13269 PMC7948698

[8] V. Nesi-Reis , D. S. S. L. Lera-Nonose , J. Oyama , M. P. P. Silva-Lalucci , I. G. Demarchi , S. M. A. Aristides , J. J. V. Teixeira , T. G. V. Silveira , M. V. C. Lonardoni , Photodiagnosis Photodyn. Ther. 2018, 21, 294. 29289704 10.1016/j.pdpdt.2017.12.015

[9] T. W. Rees , P.-Y. Ho , J. Hess , ChemBioChem 2023, 24, e202200796. 36917084 10.1002/cbic.202200796 PMC10947373

[10] B. Kar , U. Das , N. Roy , P. Paira , Coord. Chem. Rev. 2023, 474, 214860.

[11] Z. Y. Pan , D. H. Cai , L. He , Dalton Trans. 2020, 49, 11583. 32766642 10.1039/d0dt02424d

[12] A. Acosta , J. Antipán , M. Fernández , G. Prado , C. Sandoval-Altamirano , G. Günther , I. Gutiérrez-Urrutia , I. Poblete-Castro , A. Vega , N. Pizarro , RSC Adv. 2021, 11, 31959. 35495525 10.1039/d1ra06416a PMC9041655

[13] S. Zhang , M. Hosaka , T. Yoshihara , Y. Iida , K. Endo , S. Tobita , T. Takeuchi , Nat. Preced. 2007, 10.1038/npre.2007.1443.1.

[14] B. F. Hohlfeld , B. Gitter , C. J. Kingsbury , K. J. Flanagan , D. Steen , G. D. Wieland , N. Kulak , M. O. Senge , A. Wiehe , Chem. 2021, 27, 6440. 10.1002/chem.202004776 PMC8248005 33236800

[15] A. Gupta , P. Prasad , S. Gupta , P. K. Sasmal , ACS Appl. Mater. Interfaces 2020, 12, 35967. 32662979 10.1021/acsami.0c11161

[16] M. S. Capper , H. Packman , M. Rehkämper , ChemBioChem 2020, 21, 2111. 32196894 10.1002/cbic.202000117 PMC7496976

[17] A. H. Groll , B. J. A. Rijnders , T. J. Walsh , J. Adler-Moore , R. E. Lewis , R. J. M. Brüggemann , Clin. Infect. Dis. 2019, 68, S260. 31222253 10.1093/cid/ciz076 PMC6495018

[18] W. Li , X. Wu , H. Liu , C. Shi , Y. Yuan , L. Bai , X. Liao , Y. Zhang , Y. Liu , J. Inorg. Biochem. 2022, 233, 111868. 35636300 10.1016/j.jinorgbio.2022.111868

[19] N. Manav , P. E. Kesavan , M. Ishida , S. Mori , Y. Yasutake , S. Fukatsu , H. Furuta , I. Gupta , Dalton Trans. 2019, 48, 2467. 30694280 10.1039/c8dt04540b

[20] N. Manav , M. Y. Lone , M. K. Raza , J. Chavda , S. Mori , I. Gupta , Dalton Trans. 2022, 51, 3849. 35226013 10.1039/d1dt04218a

[21] J. F. Lovell , C. S. Jin , E. Huynh , H. Jin , C. Kim , J. L. Rubinstein , W. C. W. Chan , W. Cao , L. V. Wang , G. Zheng , Nat. Mater. 2011, 10, 324. 21423187 10.1038/nmat2986

[22] T. Ho , K. Guidolin , A. Makky , M. Valic , L. Ding , J. Bu , M. Zheng , M. H. Y. Cheng , J. Yau , J. Chen , G. Zheng , Angew. Chem., Int. Ed. 2023, 62, e202218218. 10.1002/anie.202218218 36811315

[23] N. Kwon , G. O. Jasinevicius , G. Kassab , L. Ding , J. Bu , L. P. Martinelli , V. G. Ferreira , A. Dhaliwal , H. H. L. Chan , Y. Mo , V. S. Bagnato , C. Kurachi , J. Chen , G. Zheng , H. H. Buzzá , Angew. Chem., Int. Ed. 2023, 62, e202305564. 10.1002/anie.202305564 37162307

[24] J. Lazniewska , C. Bader , S. M. Hickey , S. Selemidis , J. O’Leary , P. V. Simpson , S. Stagni , S. E. Plush , M. Massi , D. Brooks , Metallomics 2022, 14, mfac040. 10.1093/mtomcs/mfac040 PMC9344854 35657681

[25] P. Kusuma , P. M. Pattison , B. Bugbee , Hortic. Res. 2020, 7, 56. 32257242 10.1038/s41438-020-0283-7 PMC7105460

[26] P. Avci , A. Gupta , M. Sadasivam , D. Vecchio , Z. Pam , N. Pam , M. R. Hamblin , Semin. Cutan. Med. Surg. 2013, 32, 41. 24049929 PMC4126803

[27] M. L. Briuglia , C. Rotella , A. McFarlane , D. A. Lamprou , Drug Deliv. Transl. Res. 2015, 5, 231. 25787731 10.1007/s13346-015-0220-8

[28] L. Coderch , J. Fonollosa , M. De Pera , J. Estelrich , A. De La Maza , J. L. Parra , J. Control. Release 2000, 68, 85. 10884582 10.1016/s0168-3659(00)00240-6

[29] Y. Sadzuka , K. Kishi , S. Hirota , T. Sonobe , J. Liposome Res. 2003, 13, 157. 12855110 10.1081/lpr-120020318

[30] K. Godbout , J. P. Tremblay , Pharmaceutics 2022, 14, 2129. 36297564 10.3390/pharmaceutics14102129 PMC9611171

[31] A. F. Radovic-Moreno , T. K. Lu , V. A. Puscasu , C. J. Yoon , R. Langer , O. C. Farokhzad , ACS Nano 2012, 6, 4279. 22471841 10.1021/nn3008383 PMC3779925

[32] Z. Drulis-Kawa , A. Dorotkiewicz-Jach , J. Gubernator , G. Gula , T. Bocer , W. Doroszkiewicz , Int. J. Pharm. 2009, 367, 211. 18952159 10.1016/j.ijpharm.2008.09.043

[33] M. Pourhajibagher , H. Mahmoudi , L. Rezaei‐soufi , M. Y. Alikhani , A. Bahador , Photodiagnosis Photodyn. Ther. 2020, 30, 101717. 10.1016/j.pdpdt.2020.101717 32165339

[34] M. Mirzahosseinipour , K. Khorsandi , R. Hosseinzadeh , M. Ghazaeian , F. K. Shahidi , Photodiagnosis Photodyn. Ther. 2020, 29, 101639. 31899378 10.1016/j.pdpdt.2019.101639

[35] Z. Zhao , J. Ma , Y. Wang , Z. Xu , L. Zhao , J. Zhao , G. Hong , T. Liu , Front. Microbiol. 2021, 12, 622410. 33717010 10.3389/fmicb.2021.622410 PMC7943878

[36] H. H. Buzzá , F. Alves , A. J. B. Tomé , J. Chen , G. Kassab , J. Bu , V. S. Bagnato , G. Zheng , C. Kurachi , Proc. Natl. Acad. Sci. USA 2022, 119, e2216239119. 36346844 10.1073/pnas.2216239119 PMC9674234

