# Supplementary material for: Structural Effect of Rhenium‐ and Iridium‐Complex Liposome Composition on Their Selectivity for Antimicrobial Photodynamic Therapy
Source: Small Sci. 2023 Dec 14;4(2):2300131. doi: 10.1002/smsc.202300131 (PMC11934977; doi:10.1002/smsc.202300131)
Supplement: Supplementary file 1 — Supplementary Material [file SMSC-4-2300131-s001.pdf]

Supporting Information

Structural Effect of Rhenium- and Iridium-Complex Liposome Composition on Their Selectivity for Antimicrobial Photodynamic Therapy

Giulia Kassab+, Neha Manav+, Layla Pires, Miffy H. Y. Cheng, Yulin Mo, Layla Pires, Hilde H. Buzzá, I. Gupta, Juan Chen, Gang Zheng**

1. Synthesis of rhenium-lipid conjugate (1) and iridium-lipid conjugate

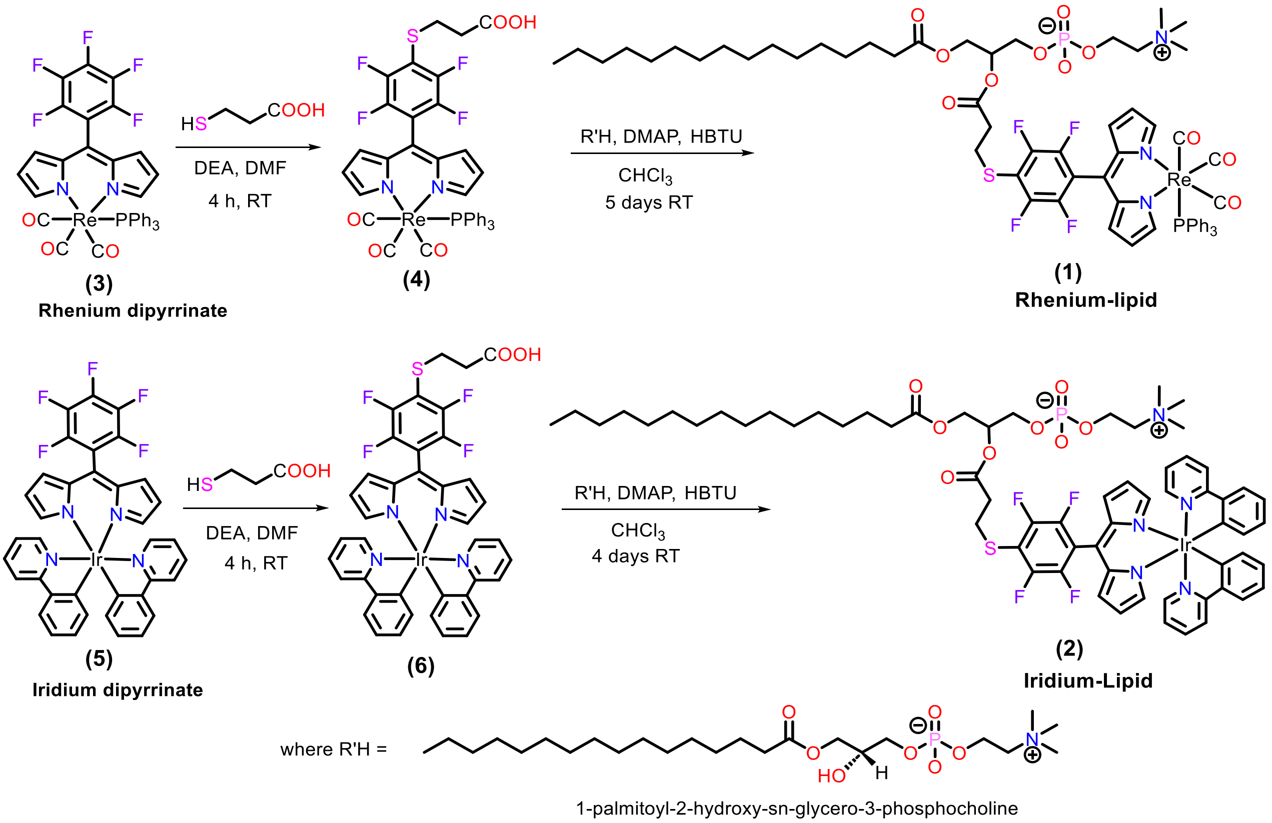

Scheme S1. Synthesis of rhenium-lipid conjugate (**1**) and iridium-lipid conjugate **(2)**.

2. Molecular characterization of rhenium dipyrrinate acid: compound (4)

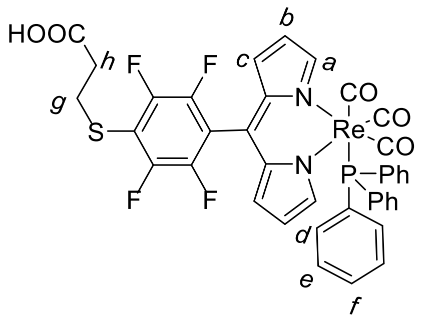

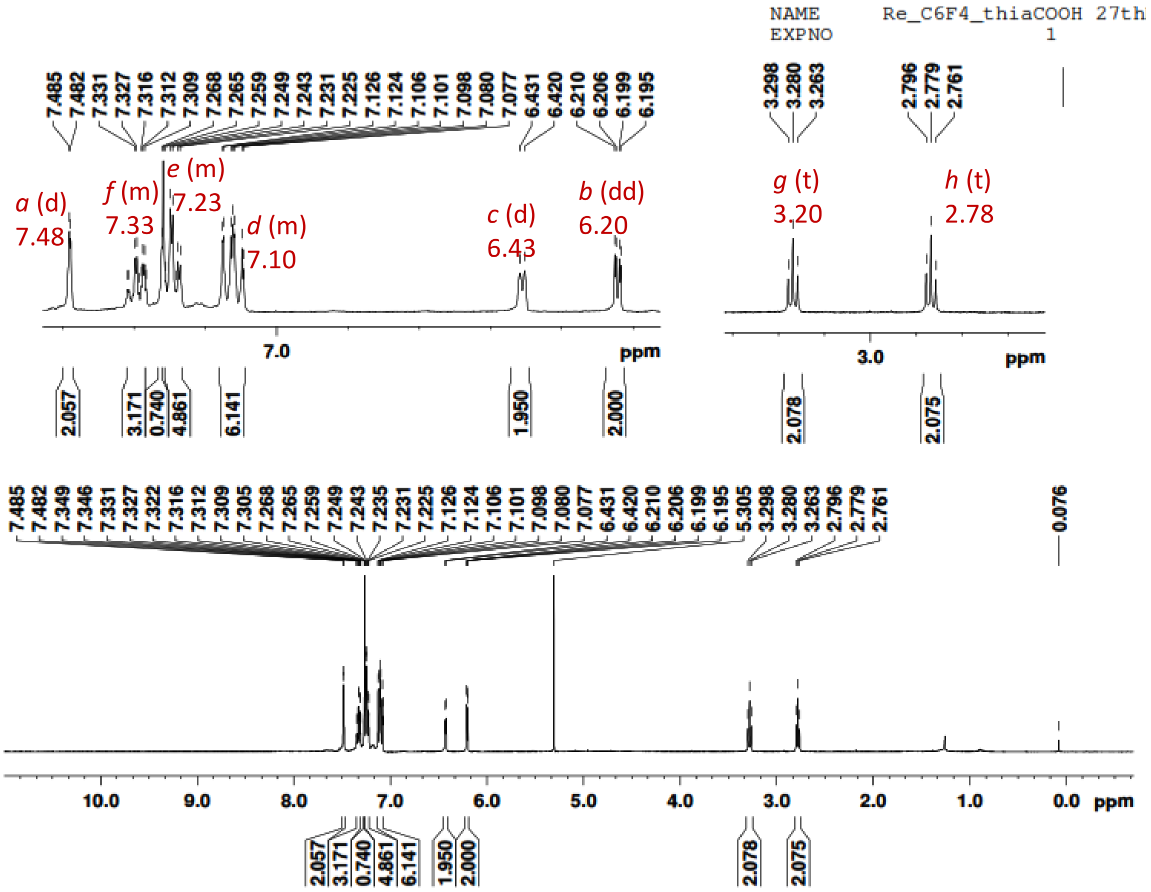

Figure S1 ^{1}H NMR spectrum of rhenium dipyrrinate acid, compound (**4**) in $CDCl_3$.

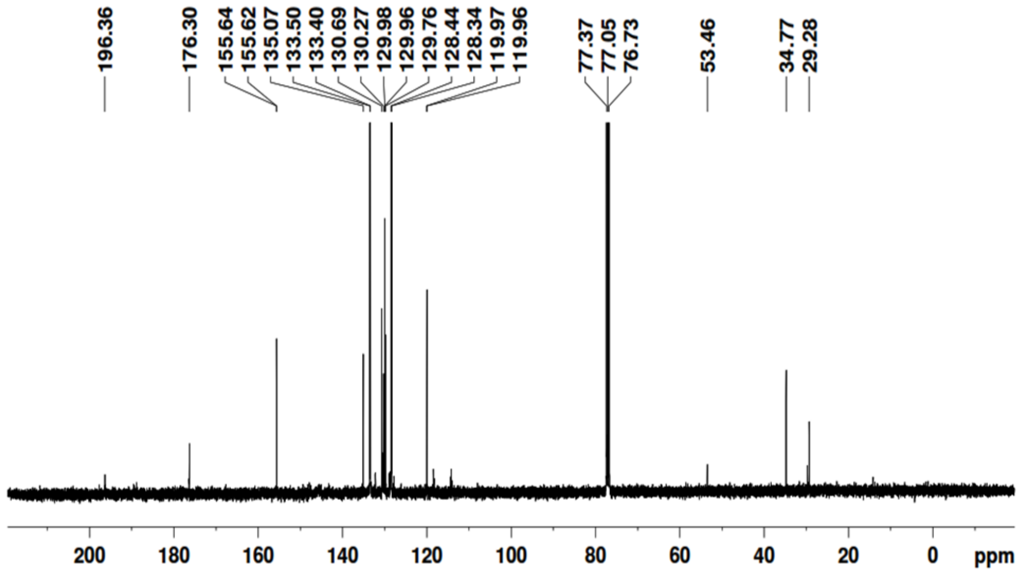

Figure S2. The ^{13}C NMR of compound 4 in $CDCl_3$.

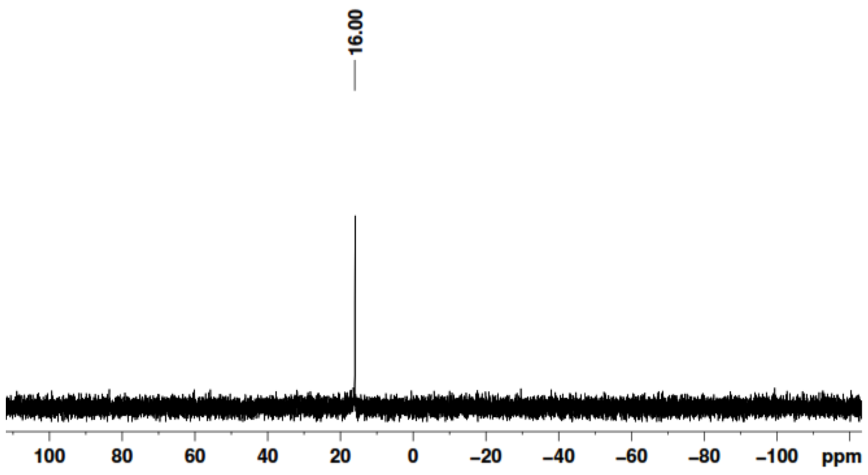

Figure S3. The ^{31}P NMR of rhenium dipyrrinate acid, compound (**4**) in $CDCl_3$.

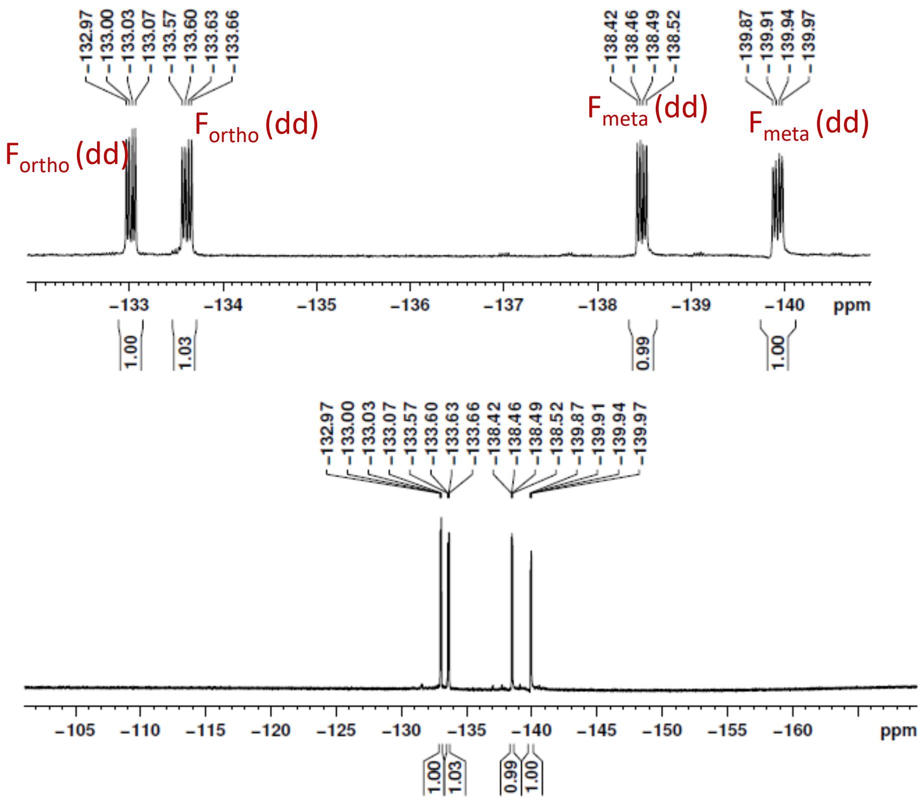

Figure S4. The ^{19}F NMR of rhenium dipyrrinate acid, compound (**4**) in $CDCl_3$.

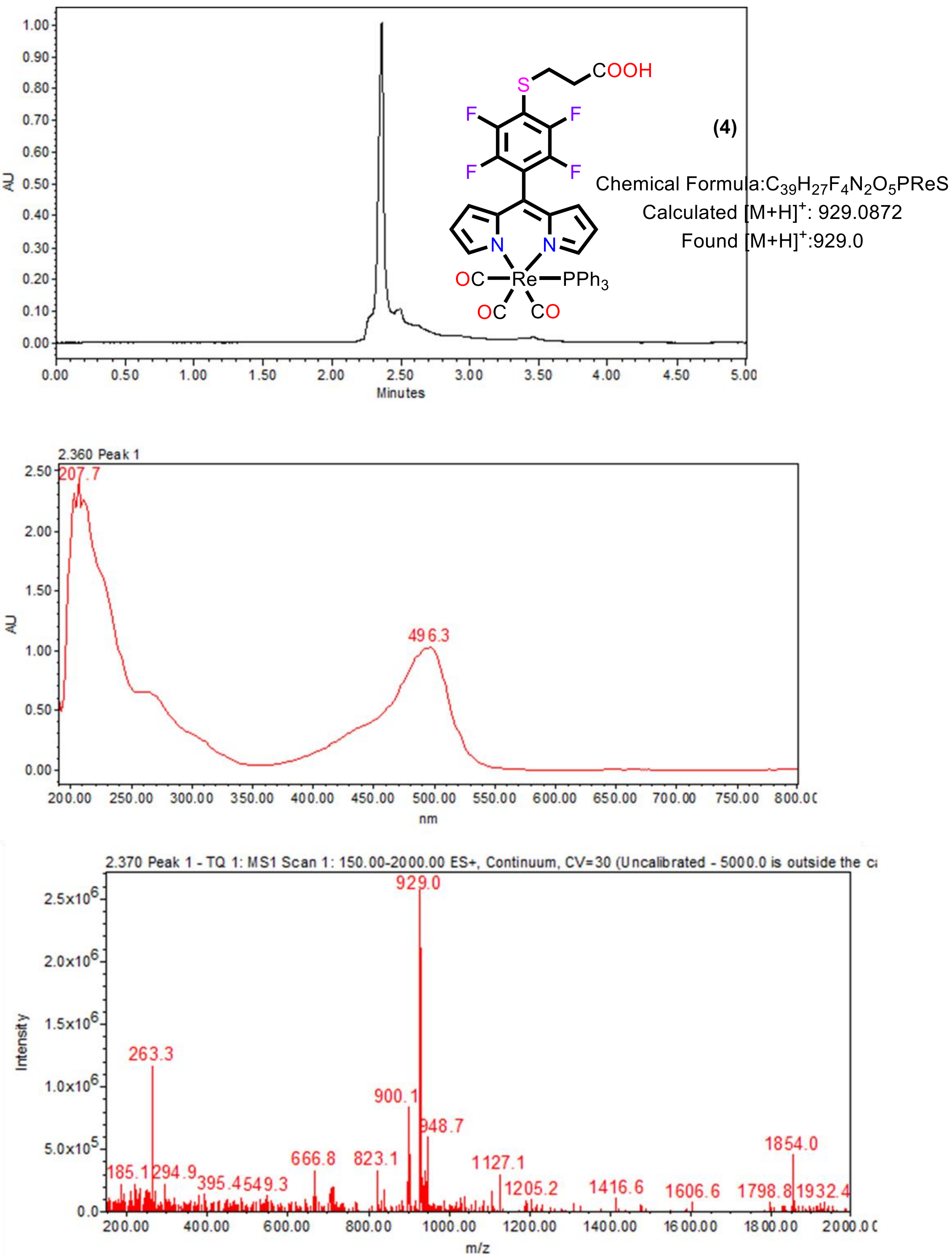

Figure S5. The UPLC-chromatogram, associated UV-visible and ESI-MS spectra of rhenium dipyrrinate acid: compound (**4**).

3. Molecular characterization of Iridium dipyrrinate acid: compound (6)

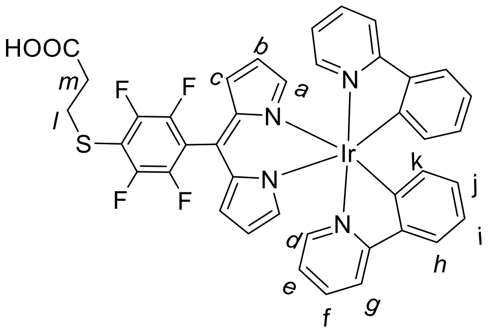

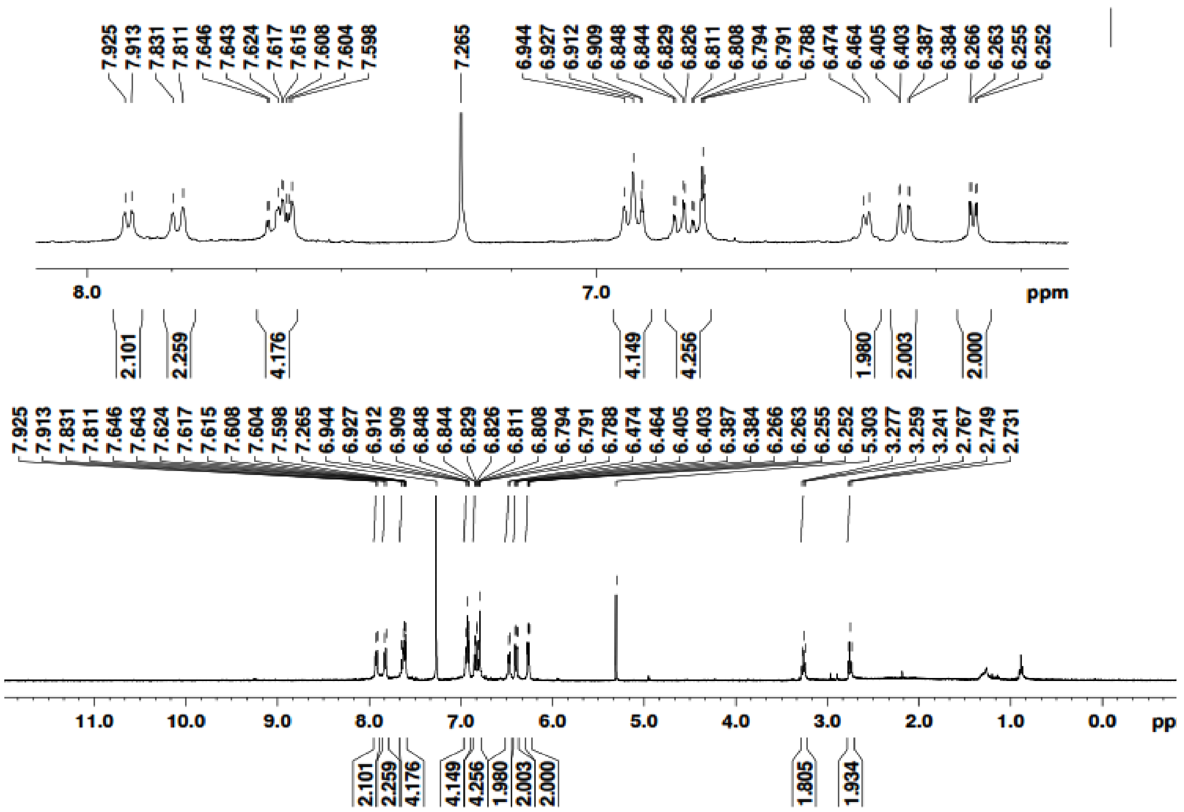

Figure S6 ^{1}H NMR spectrum of Iridium dipyrrinate acid, compound (**6**) in $CDCl_3$.

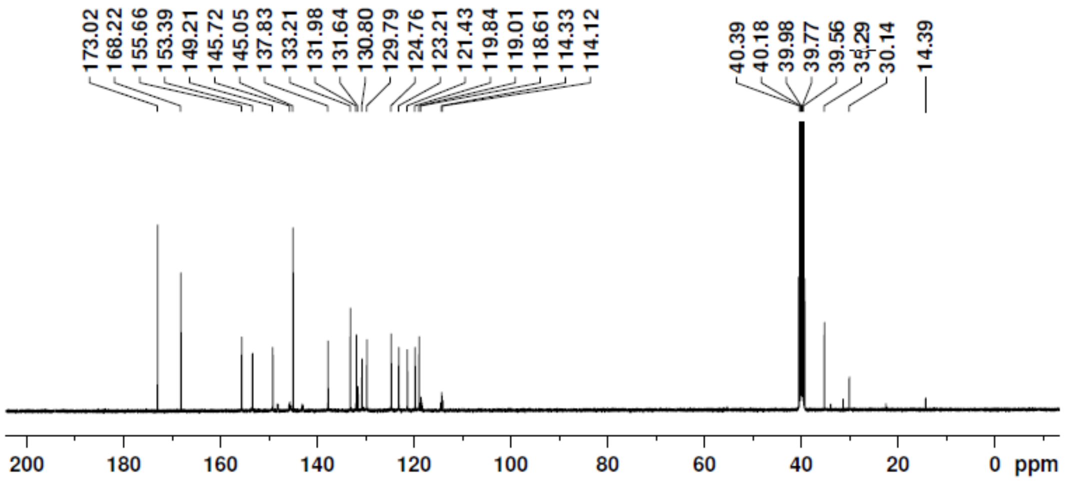

Figure S7. The ^{13}C NMR of Iridium dipyrrinate acid, compound (**6**) in DMSO.

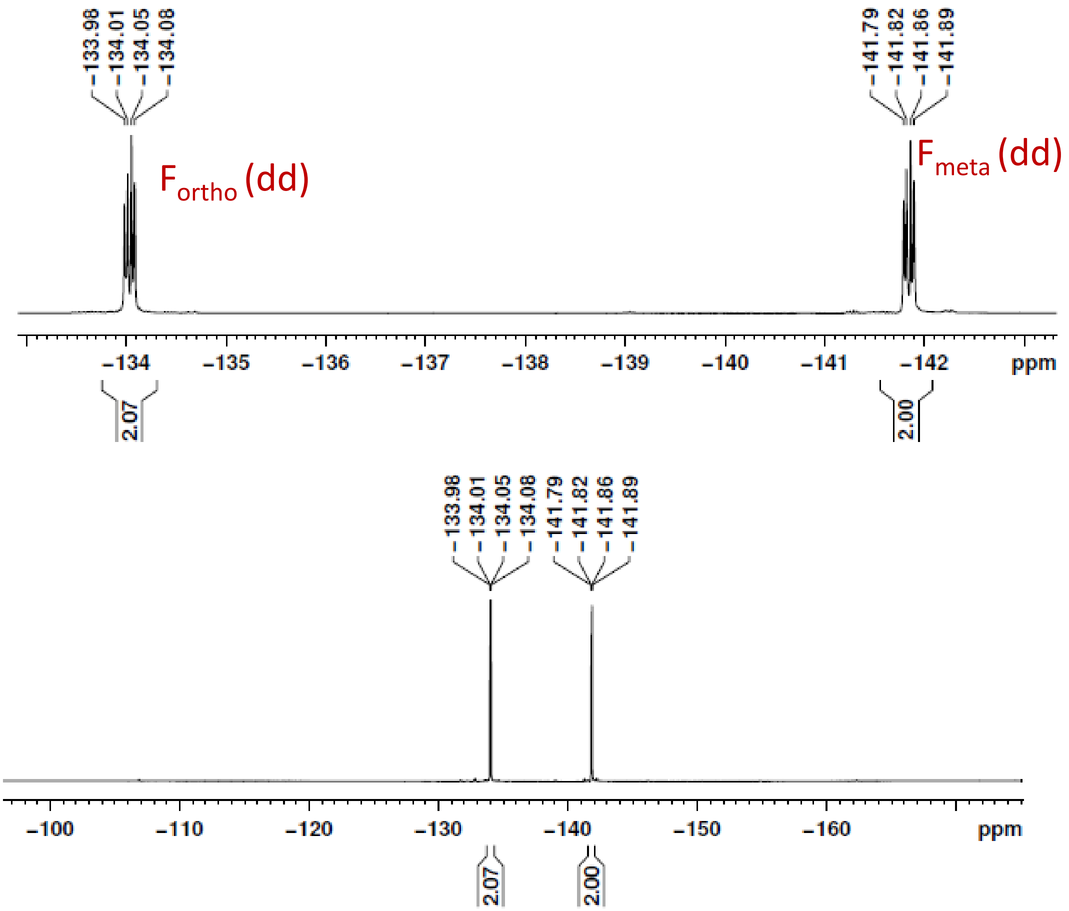

Figure S8. The ^{19}F NMR of Iridium dipyrrinate acid, compound (**6**) in DMSO.

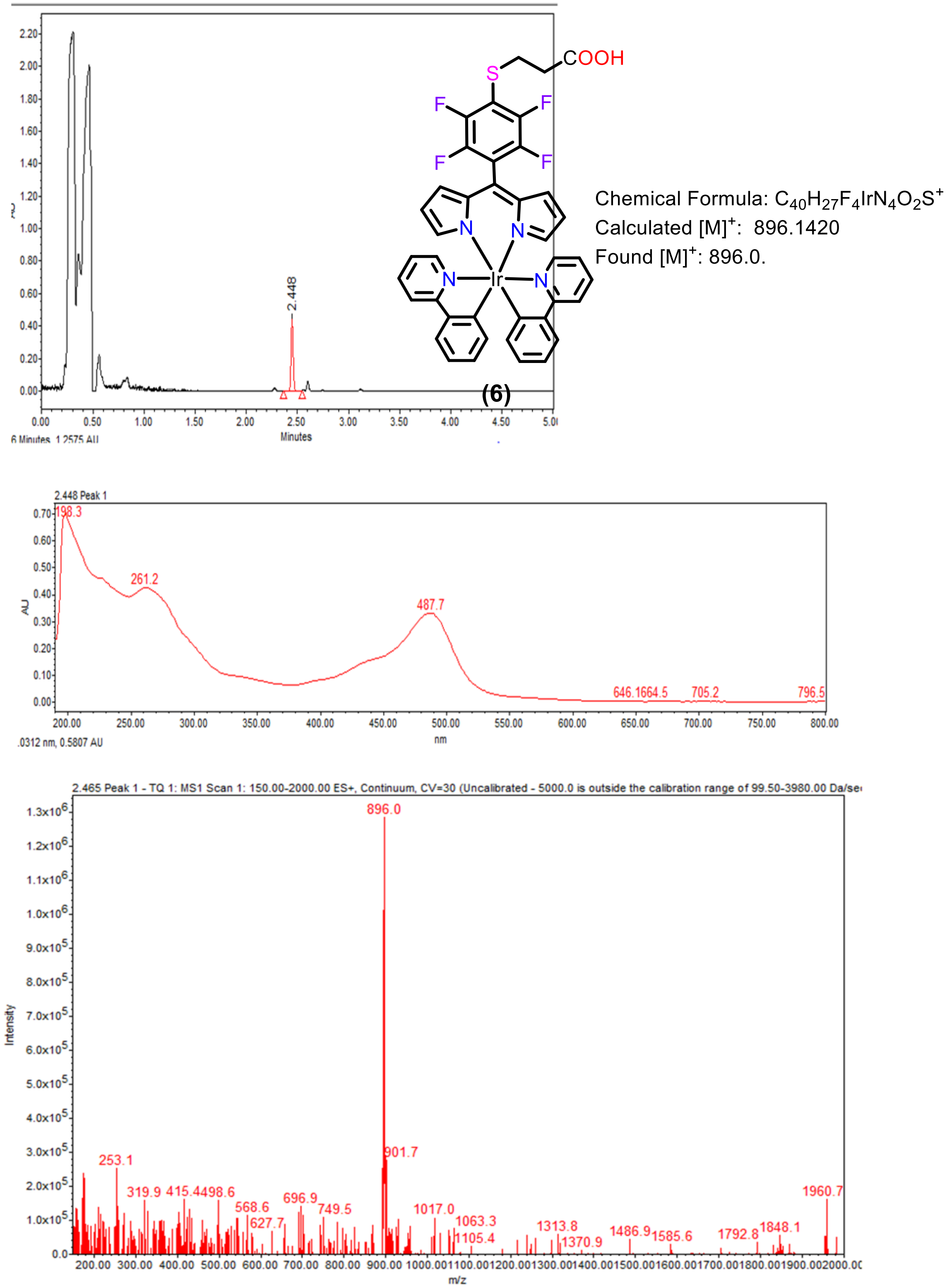

Figure S9. The UPLC-chromatogram, associated UV-visible and ESI-MS spectra of iridium dipyrrinate acid: compound (**6**).

4. **Molecular characterization of rhenium complex lipid conjugate (Re-lipid): compound (1)**

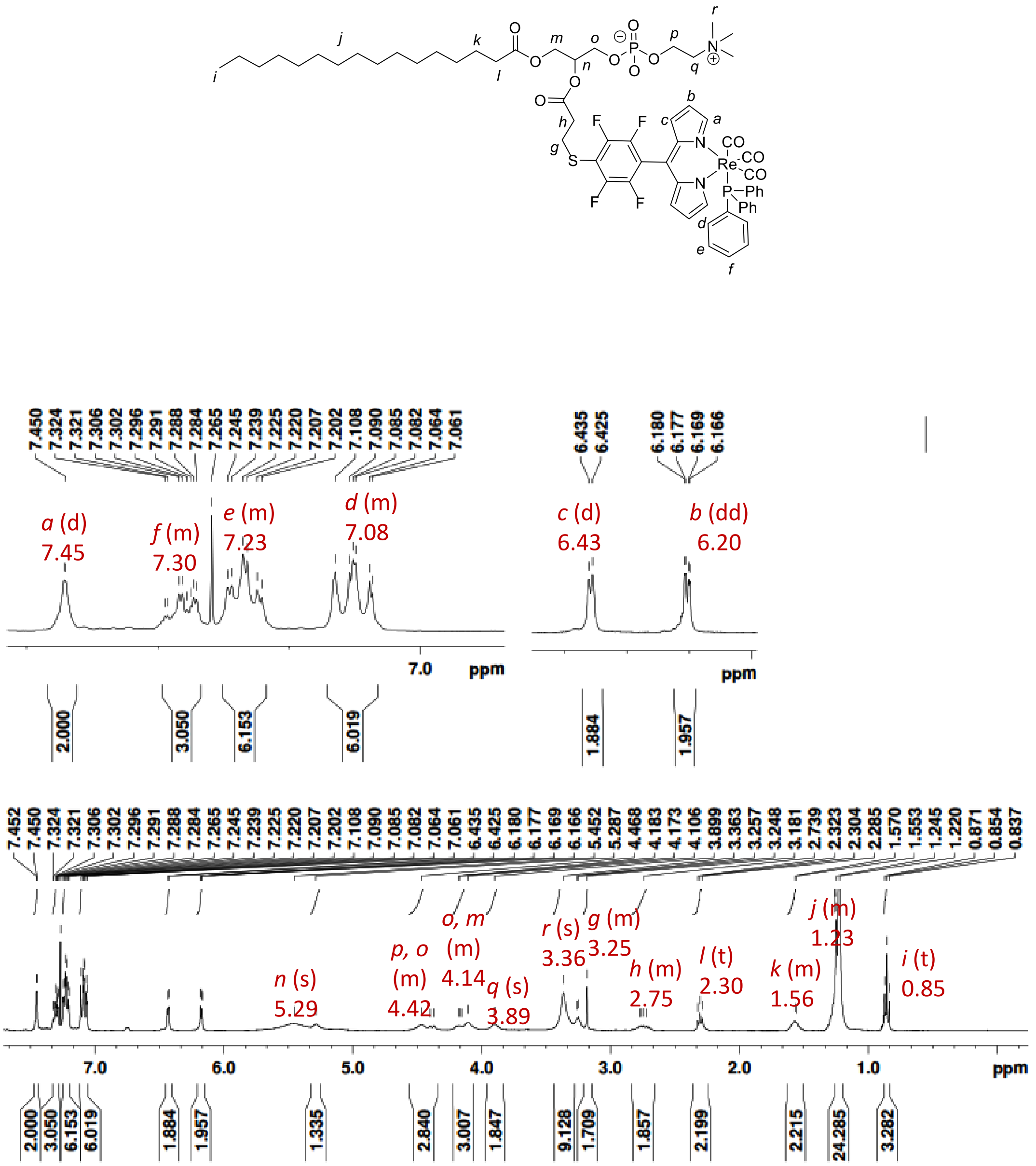

Figure S10. The ^{1}H NMR of Re-lipid, compound (**1**) in $CDCl_3$.

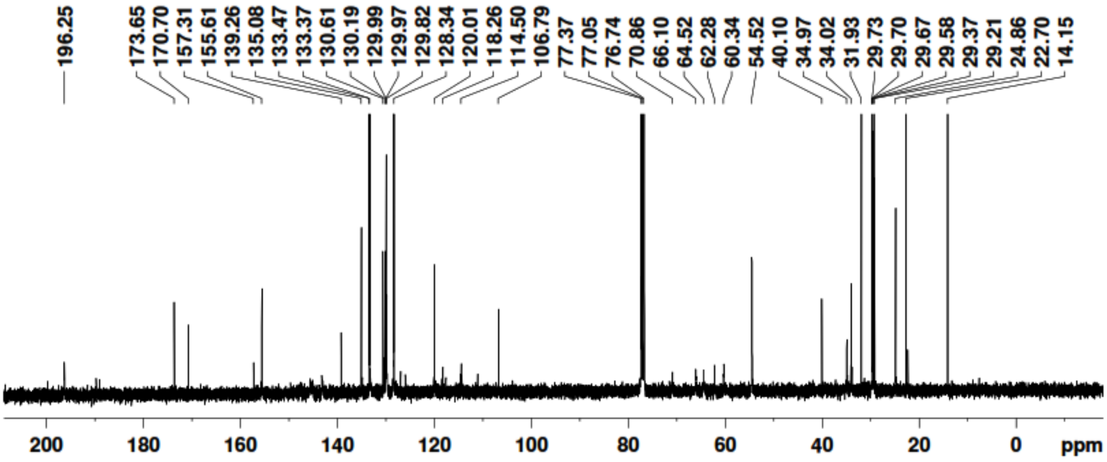

Figure S11. The ^{13}C NMR of Re-lipid, compound (**1**) in $CDCl_3$.

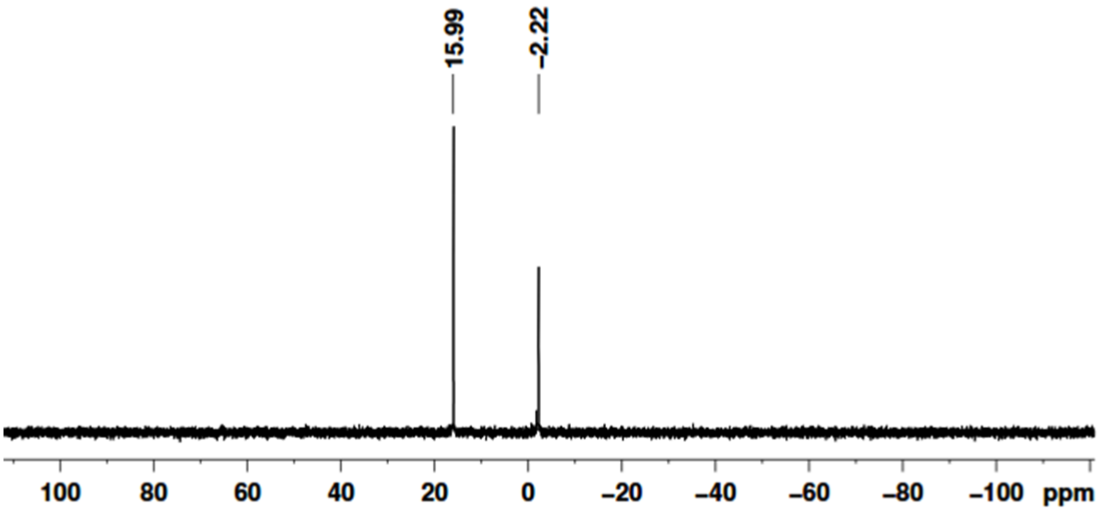

Figure S12. The ^{31}P NMR of Re-lipid, compound (**1**) in $CDCl_3$.

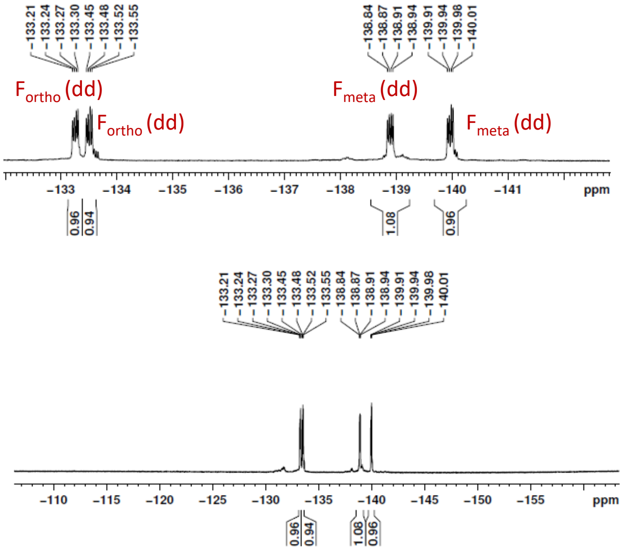

Figure S13. The ^{19}F NMR of Re-lipid, compound (1) in $CDCl_3$.

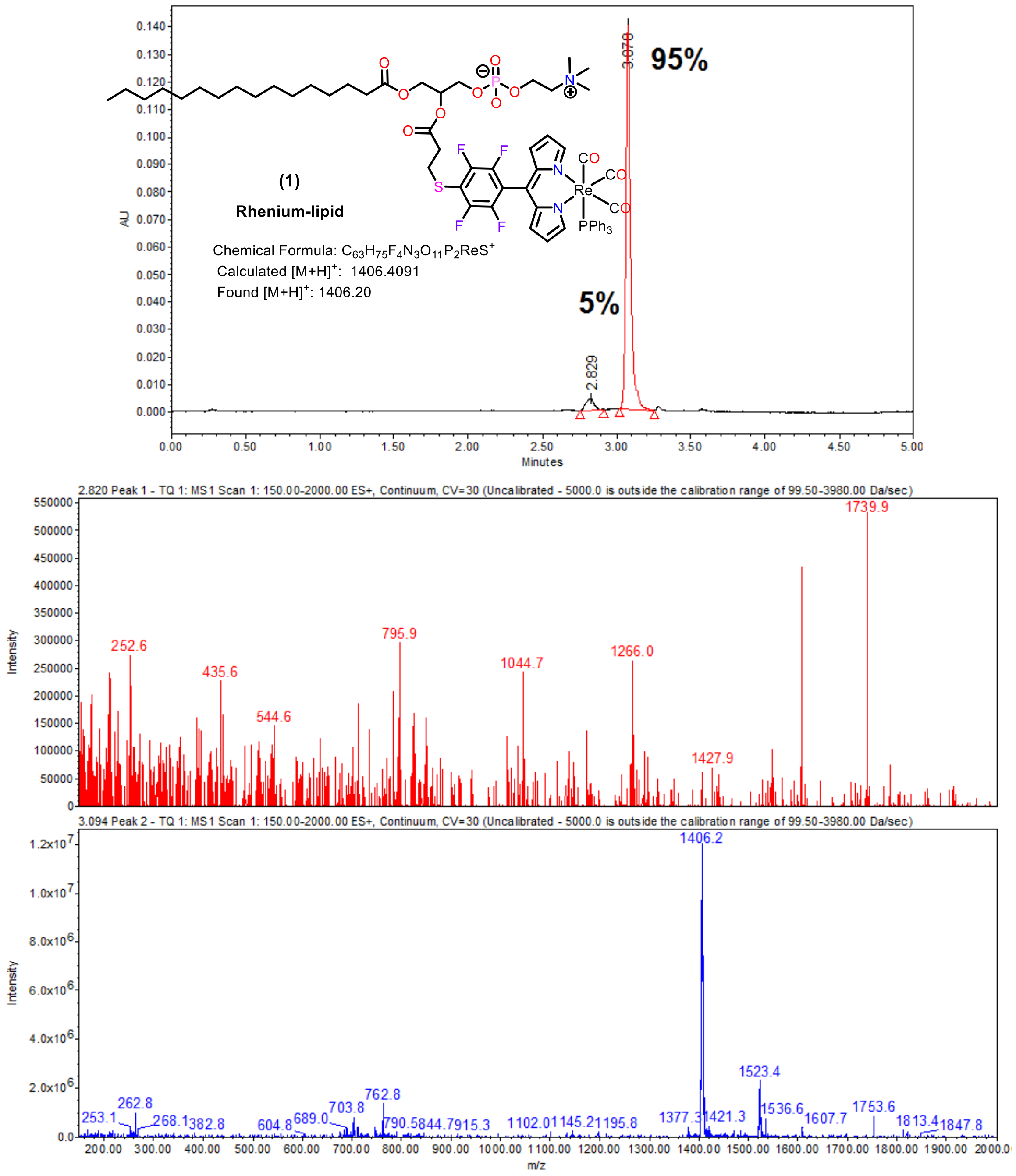

Figure S14. The UPLC-chromatogram and ESI-MS spectra of rhenium-lipid: compound (**1**).

5. Molecular characterization of Iridium complex lipid conjugate (Ir-lipid): compound (2)

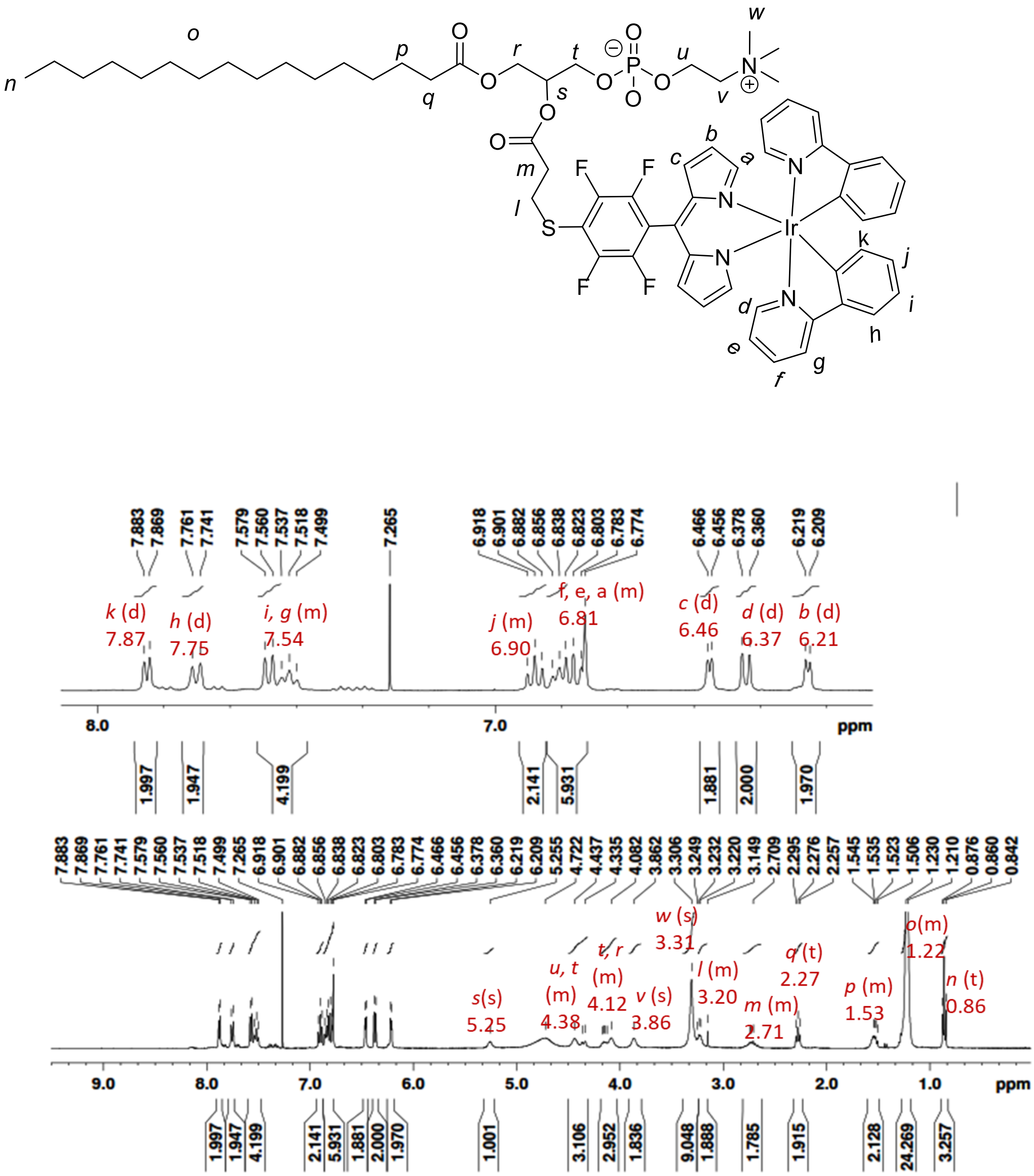

Figure S15. The ^{1}H NMR of Ir-lipid, compound (**2**) in $CDCl_3$.

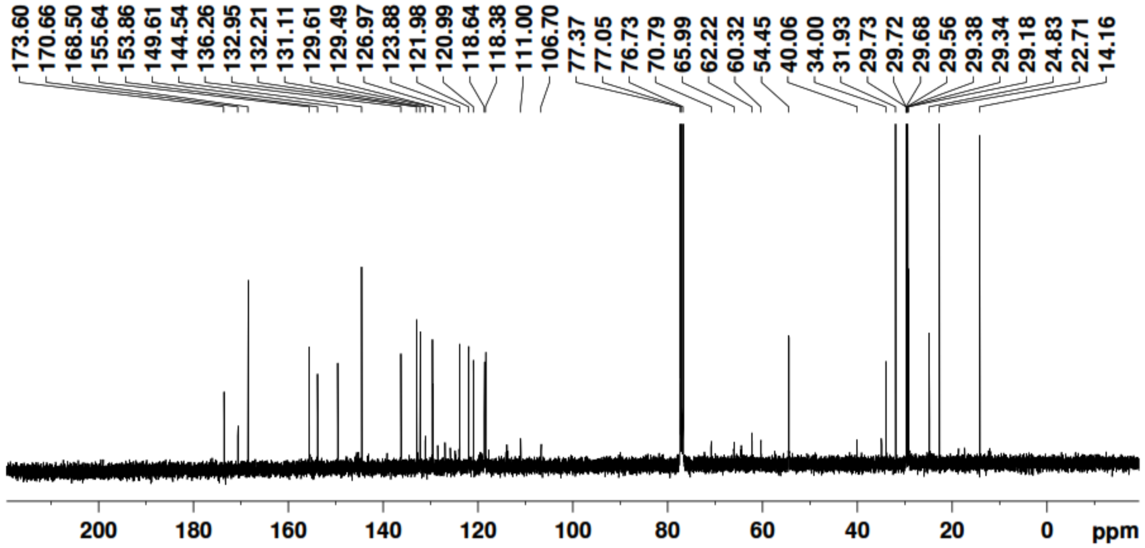

Figure S16. The ^{13}C NMR of Ir-lipid, compound (**2**) in $CDCl_3$.

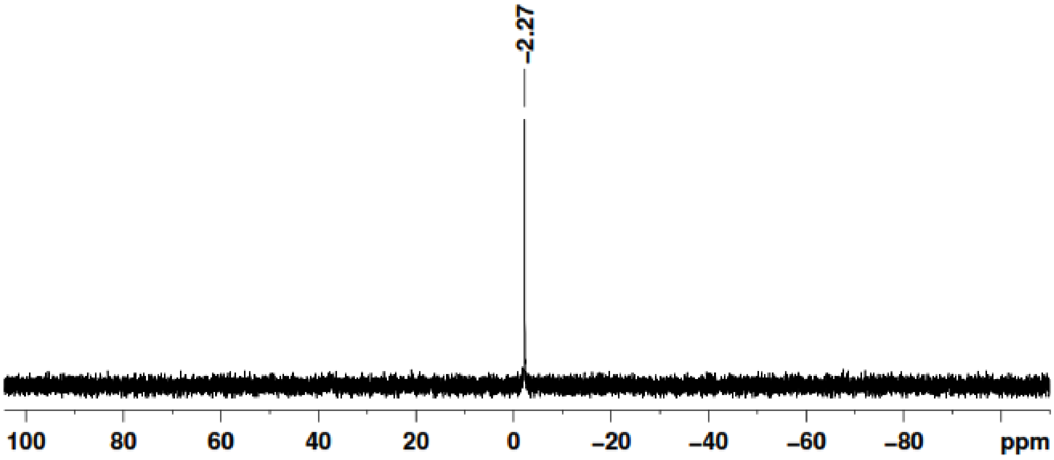

Figure S17. The ^{31}PNMR of compound 2 (Ir-lipid) in $CDCl_3$.

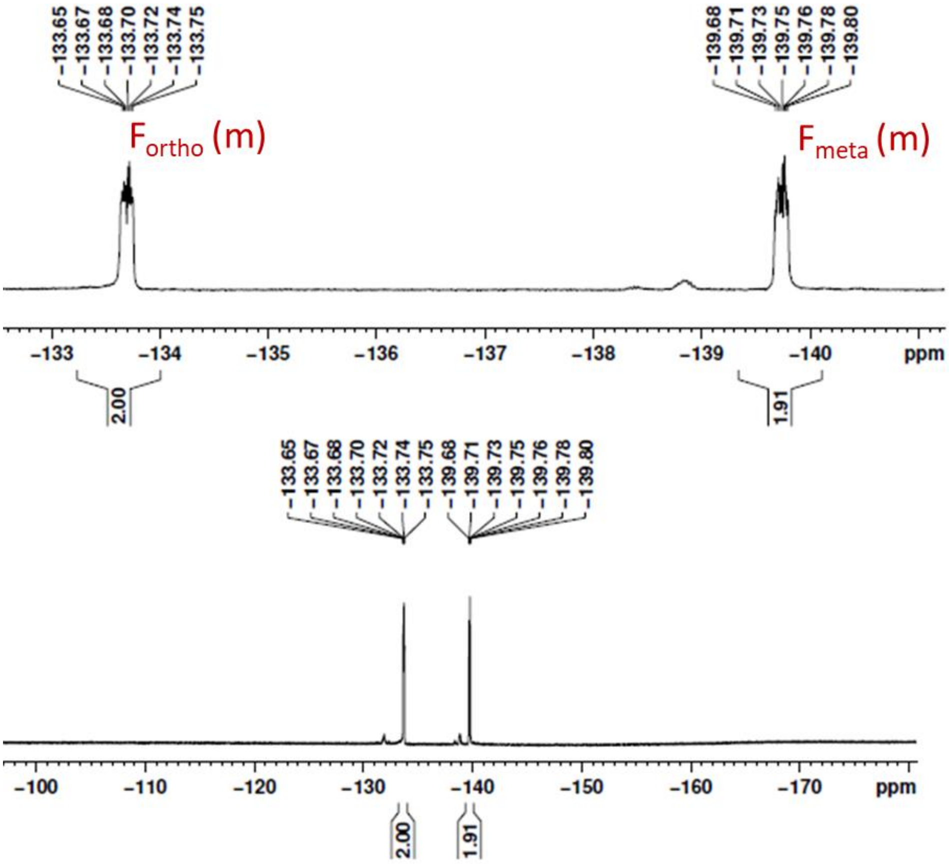

Figure S18. The ^{19}F NMR of Ir-lipid, compound (**2**) in $CDCl_3$.

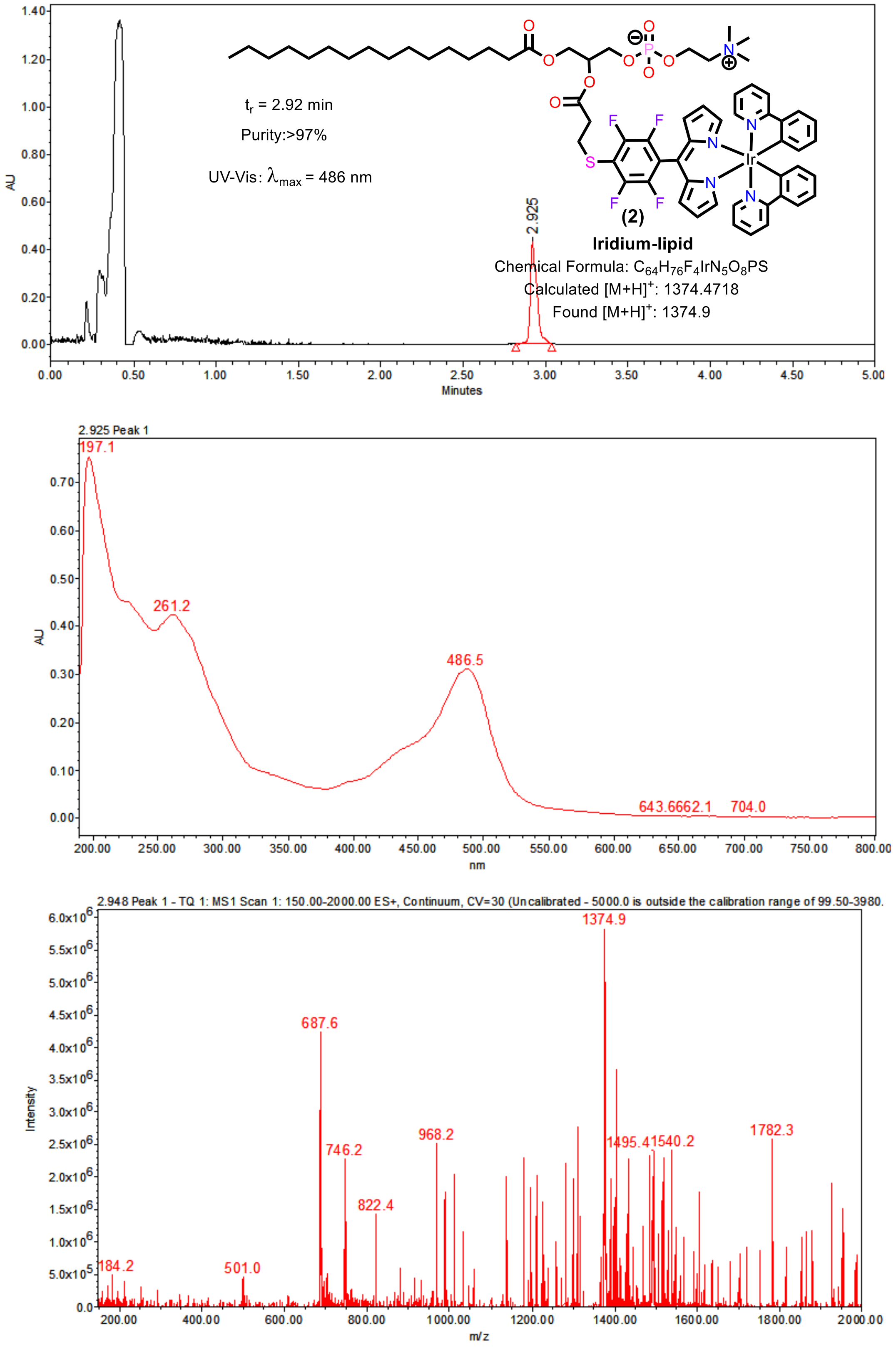

Figure S19. The UPLC-chromatogram, associated UV-visible and ESI-MS spectra of iridium lipid: compound **(2)** (Ir-lipid).

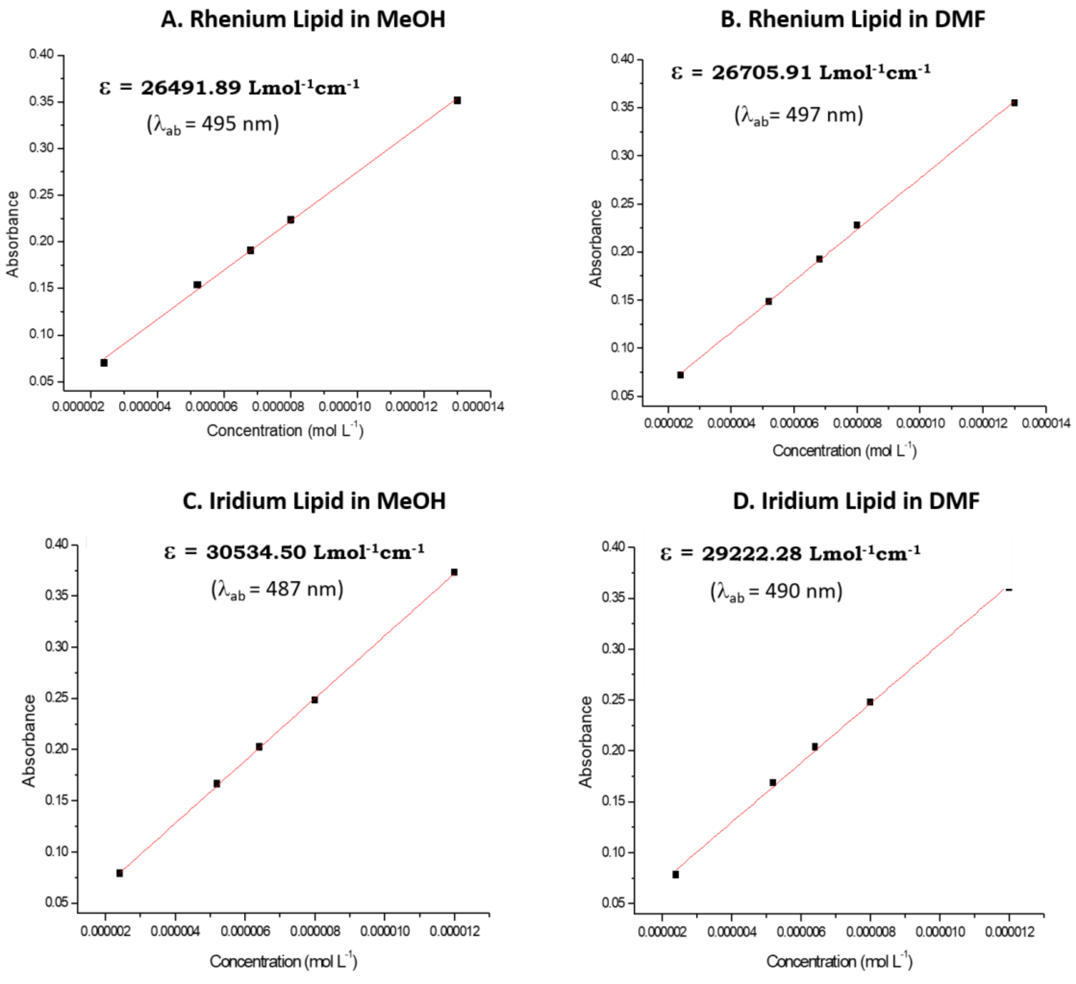

Figure S20. Absorbance at maximum peak and calculated extinction coefficient of Re-lipid and Ir-lipid in Methanol and DMF.

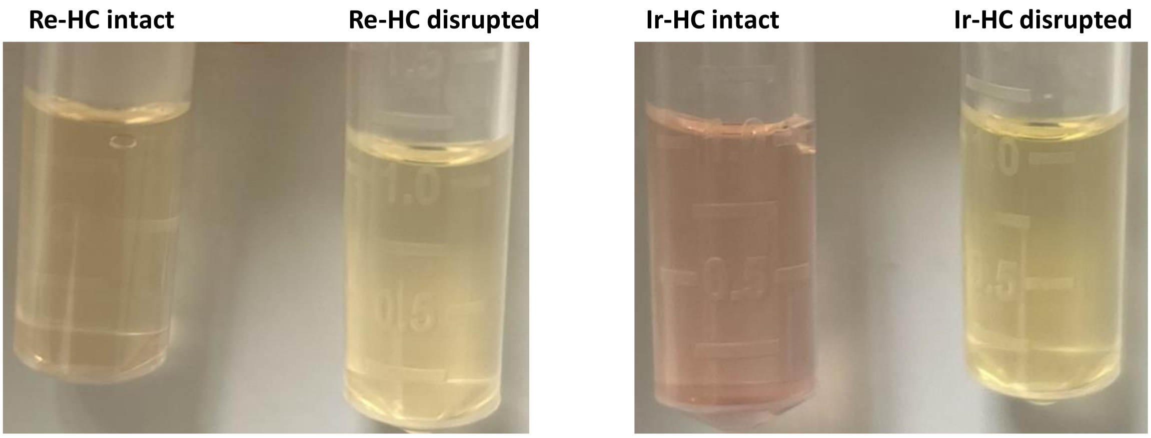

Figure S21. Photographs of intact and disrupted Re-HC (10μM) and Ir-HC (10μM) in solution.

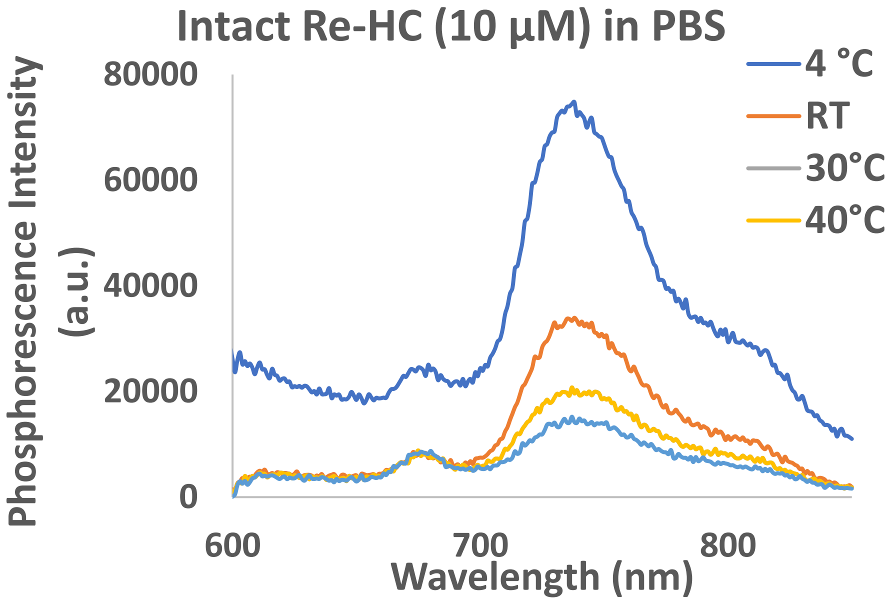

Figure **S22**. The Re-HC emission intensity exhibited a decrease with rising temperature.

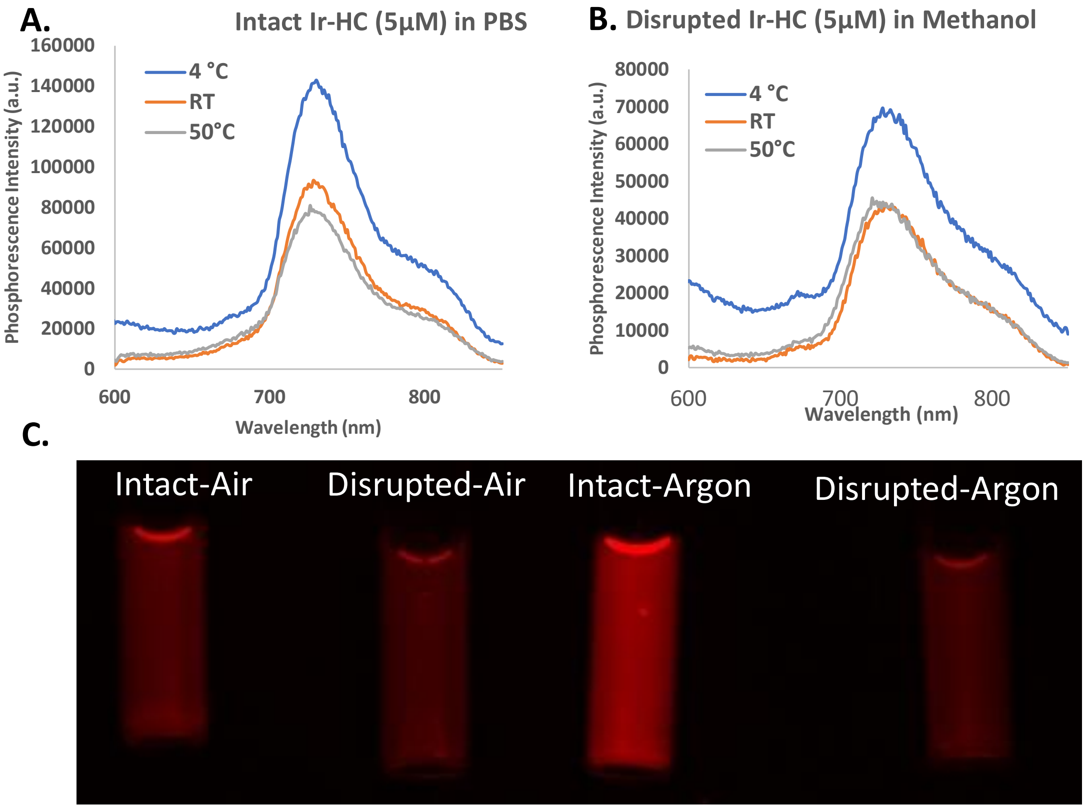

Figure S23. The Ir-HC emission intensity decreased with rising temperature in both nanoparticles A) intact (PBS) and B) disrupted forms (Methanol); C) Ir-HC generated stronger emission in an inert gas (Argon) environment compared to under ambient air conditions (Ir-HC 50µM concentration, Green Ex. Filter 500-560 nm; Red Em Filter 700-800 nm; Exposure time: 500 ms.

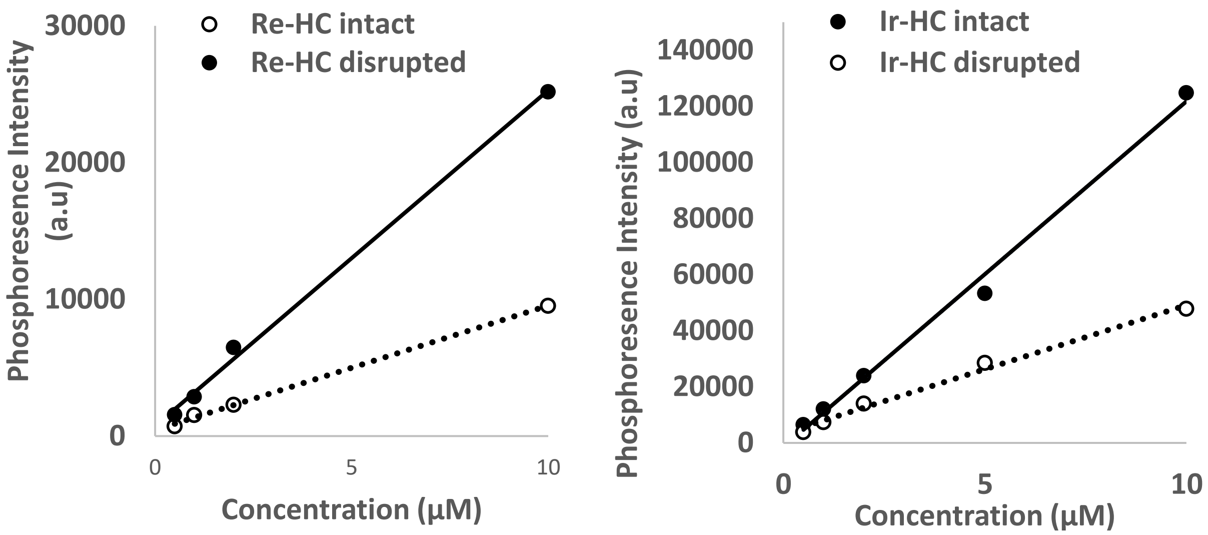

Figure S24. Re-HC and Ir-HC emitted phosphorescence shows a strong correlation with their concentration in both intact and disrupted forms under 495 nm excitation.

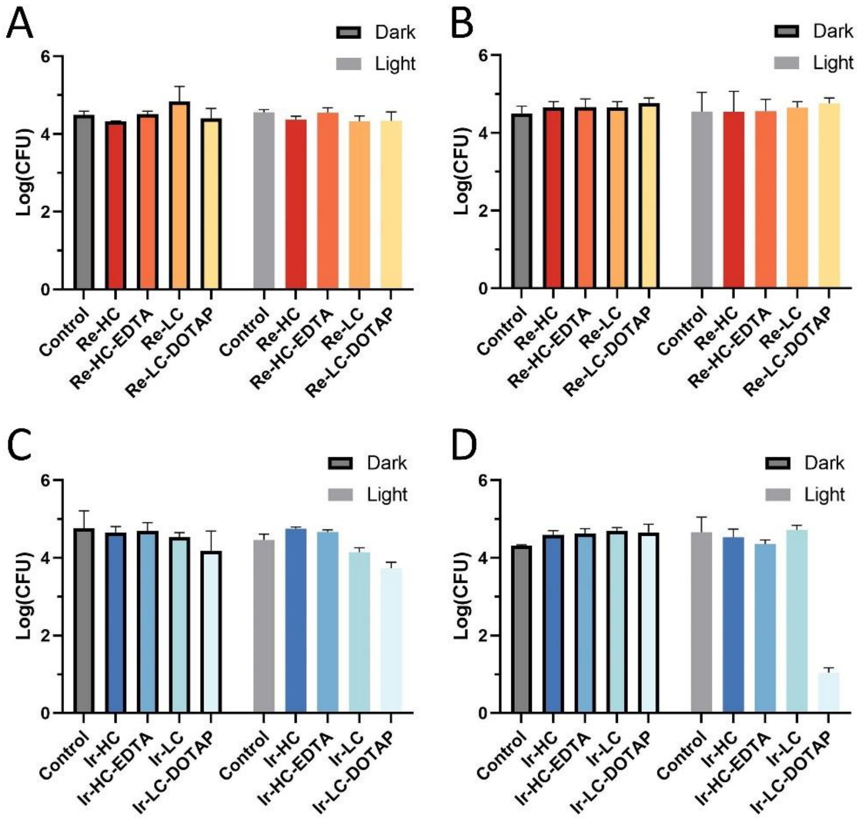

Figure S25. Effect of the Rhenium (A, B) and Iridium (C, D) liposomal formulations on the viability of *E. coli* with and without light treatment. A and C: 10 µM of active lipid, 15 minutes of drug-light interval, and 30 J/cm^2 of 450 nm at 70 mW/cm^2 for light-treated groups. B and D: 50 µM of active lipid, 60 minutes of drug-light interval, and 60 J/cm^2 of 450 nm at 70 mW/cm^2 for light-treated groups. N=3.

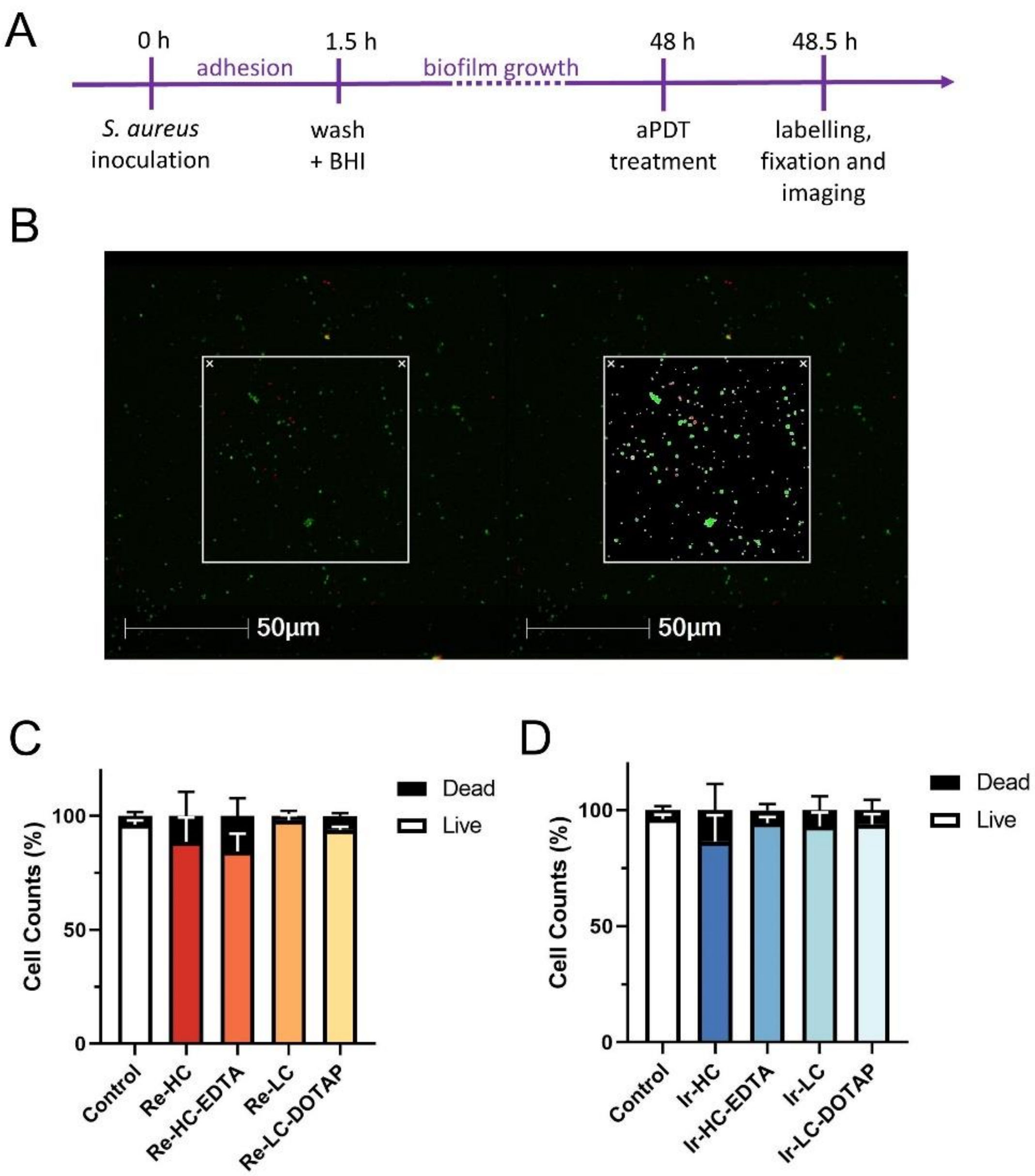

Figure S26. Experiments on *S. aureus* biofilm. A: schematic representation of the experimental timeline: chamber slides were inoculated with *S. aureus* in PBS in a rocking incubator (37 °C, 30 rpm, 8° tilt), washed and filled with BHI for biofilm formation. The same aPDT treatment from other experiments was performed: 10 µM of active lipid, 15 minutes DLI, and 30 J/cm². Labelling was performed with FilmTracer LIVE/DEAD (Invitrogen), and imaging was done with confocal microscopy. B: Representative confocal image of a sample (600X) and live/dead delimitation from HALO analysis (Indica Labs). C and D: Live and dead cell counts after aPDT with the Rhenium (C) and Iridium (D) formulations. N = 3.
